# Topological Reorganization of Odor Representations in the Olfactory Bulb

**DOI:** 10.1371/journal.pbio.0050178

**Published:** 2007-07-03

**Authors:** Emre Yaksi, Benjamin Judkewitz, Rainer W Friedrich

**Affiliations:** 1 Department of Biomedical Optics, Max-Planck-Institute for Medical Research, Heidelberg, Germany; 2 Friedrich-Miescher-Institute, Basel, Switzerland; University of Maryland, United States of America

## Abstract

Odors are initially represented in the olfactory bulb (OB) by patterns of sensory input across the array of glomeruli. Although activated glomeruli are often widely distributed, glomeruli responding to stimuli sharing molecular features tend to be loosely clustered and thus establish a fractured chemotopic map. Neuronal circuits in the OB transform glomerular patterns of sensory input into spatiotemporal patterns of output activity and thereby extract information about a stimulus. It is, however, unknown whether the chemotopic spatial organization of glomerular inputs is maintained during these computations. To explore this issue, we measured spatiotemporal patterns of odor-evoked activity across thousands of individual neurons in the zebrafish OB by temporally deconvolved two-photon Ca^2+^ imaging. Mitral cells and interneurons were distinguished by transgenic markers and exhibited different response selectivities. Shortly after response onset, activity patterns exhibited foci of activity associated with certain chemical features throughout all layers. During the subsequent few hundred milliseconds, however, MC activity was locally sparsened within the initial foci in an odor-specific manner. As a consequence, chemotopic maps disappeared and activity patterns became more informative about precise odor identity. Hence, chemotopic maps of glomerular input activity are initially transmitted to OB outputs, but not maintained during pattern processing. Nevertheless, transient chemotopic maps may support neuronal computations by establishing important synaptic interactions within the circuit. These results provide insights into the functional topology of neural activity patterns and its potential role in circuit function.

## Author Summary

Many sensory brain areas contain topographic maps where the physical location of neuronal activity contains information about a stimulus feature. In the first central processing center of the olfactory pathway, the olfactory bulb, chemically distinct odors often elicit spatially segregated input activity so that general chemical features are initially represented in a topographic fashion. It is, however, unclear whether this “chemotopic” organization of odor representations is maintained at subsequent stages of odor processing. To address this question, we visualized activity patterns across thousands of individual neurons in the intact olfactory bulb of zebrafish over time using two-photon calcium imaging. Our results demonstrate that odor-evoked activity across the output neurons of the olfactory bulb is chemotopically organized shortly after stimulus onset but becomes more widely distributed during the subsequent few hundred milliseconds of the response. This reorganization of olfactory bulb output activity is most likely mediated by inhibitory feedback and reduces the redundancy in activity patterns evoked by related stimuli. These results indicate that topographically organized activity maps in the olfactory bulb are not maintained during information processing, but contribute to the function of local circuits.

## Abstract 3

Two-photon calcium imaging in the zebrafish olfactory bulb reveals that mitral cells show more selective responses to odors than interneurons, and odor-evoked firing patterns of populations of mitral cells evolve over hundreds of milliseconds to become more distinct for different odors, thus providing more information about odor identity.

## Introduction

The brain continuously processes patterns of sensory input to extract relevant information about the environment. In most or even all sensory systems, afferent activity patterns exhibit a topological organization in which defined stimulus variables are mapped along spatial coordinates in the brain. A continuous change in a stimulus variable therefore results in a continuous shift of activity along a distinct trajectory on the brain surface. Topological sensory maps are often elaborated during successive stages of processing [1,2] and are assumed to play important roles for information processing although their precise functions are not well defined [3]. Odors differ from most other sensory stimuli because they span a high-dimensional stimulus space and cannot be described by a small number of continuous variables [4,5]. It is therefore debated whether a systematic mapping of chemical stimulus features, referred to as “chemotopy,” plays a role in the processing of odor-encoding activity patterns in the brain.

Odor information is conveyed from the nose to the first central processing center, the olfactory bulb (OB), by specific patterns of activation across the array of glomeruli [6–13]. Each glomerulus receives convergent input from sensory neurons expressing the same odorant receptor [14]. As a consequence, the array of glomeruli constitutes a discrete spatial map of odorant receptor expression. Because individual odorant receptors respond to overlapping sets of molecules [5,15,16], glomerular activity patterns are combinatorial and spatially distributed. However, odors sharing certain molecular properties preferentially activate glomeruli within defined areas, as shown in various vertebrate species [9–13,17–20]. The gross functional organization of glomerular activity patterns is therefore described as a “chemotopic map” because molecular stimulus features are associated with glomerular activity in defined regions. In rodents and zebrafish, primary molecular properties (e.g., characteristic functional groups) are mapped onto relatively large domains, whereas secondary molecular features (e.g., chain length) are mapped onto subregions within these domains [9,10,12,13,17]. Chemotopic maps are therefore organized hierarchically such that “fine” maps of secondary features are nested within “coarse” maps of primary features. Within a given region, however, not all glomeruli respond to all stimuli with the associated feature, and a given stimulus usually activates glomeruli in more than one region. Compared to topological maps in other sensory systems, the chemotopy of glomerular activity patterns therefore appears rough and fractured, possibly as a consequence of reducing a high-dimensional molecular feature space onto two spatial dimensions [13,21].

Although chemotopic activity patterns have been described at the level of glomeruli, much less is known about the topological organization of odor-evoked activity patterns downstream of glomerular afferents. Patterns of glomerular input activity are processed in the OB by neuronal circuits that are conserved within vertebrates [22,23] and similar to the first olfactory processing center in insects, the antennal lobe [4]. The principal neurons of the OB, the mitral cells (MCs), receive direct excitatory input from sensory neurons and convey the output of the OB to higher brain regions. In addition, the OB contains different types of interneurons (INs) including periglomerular and granule cells. Most INs are GABAergic and mediate inhibitory lateral interactions between MCs over a wide range of spatial scales [24–26]. These network interactions create temporal patterns of activity on multiple time scales and result in dynamic changes of OB output activity patterns during an odor response [27–34]. It is, however, unclear how this circuitry processes chemotopically organized patterns of glomerular input.

A detailed analysis of topological activity patterns downstream of glomeruli requires recordings of odor responses from a substantial fraction of MCs and INs to a representative panel of stimuli with high spatial and temporal resolution. Single-unit extracellular recordings from anesthetized rabbits revealed that MCs within certain regions of the OB tend to respond to molecules sharing chemical features, suggesting that MC activity patterns exhibit at least some chemotopy [35,36]. However, the dynamics of MC activity patterns over time have not been investigated, and the spatial resolution and sample size obtained with this approach are necessarily limited. Moreover, electrophysiological recordings in mice indicate that MC activity patterns are influenced substantially by anesthesia [37]. The visualization of odor-evoked activity by 2-deoxyglucose uptake or immediate early gene expression suggests that the activation of small groups of glomeruli is associated with spatially restricted activity in deeper layers of the OB [6,11,38–41]. The comparison of 2-deoxyglucose uptake patterns from multiple animals, each stimulated with one odor, could provide information about chemotopic maps, but has been performed only for the glomerular layer [11]. Moreover, this technique has relatively low spatial resolution and lacks temporal resolution. Patterns evoked by multiple stimuli within the same OB have been visualized by functional magnetic resonance imaging to study chemotopic maps in the glomerular layer of rodents [20,42]. Signals in deeper layers, however, appeared weak and have not been analyzed. Optical imaging techniques appear promising but cannot penetrate deep into the OB and provide only a limited field of view. In the salamander, odor-evoked activity patterns throughout multiple layers have been measured by voltage-sensitive dye imaging [43], but chemotopy has not been studied systematically. Results obtained with different methods therefore indicate that odor-evoked activity across MCs and INs is not uniformly distributed but have not enabled a systematic analysis of chemotopy.

Activity patterns across large numbers of individual neurons in the intact vertebrate brain have recently been measured by two-photon Ca^2+^ imaging [44,45] after bolus loading of a Ca^2+^ indicator [46–50]. The temporal resolution of raw Ca^2+^ signals is limited by the kinetics of unitary somatic Ca^2+^ transients. However, changes in firing rates can be reconstructed from somatic Ca^2+^ signals by temporal deconvolution with a kernel representing a unitary Ca^2+^ transient [51]. This technique, referred to as temporally deconvolved Ca^2+^ imaging (TDCa imaging), substantially increases the effective temporal resolution of Ca^2+^-based activity measurements and facilitates their interpretation in terms of neural activity. Here, we used TDCa imaging to analyze the topological organization of odor-evoked activity patterns across MCs and INs during pattern processing in the OB of zebrafish. The zebrafish is an attractive model system because the architecture of neuronal circuits in the OB is similar to other vertebrates, but contains fewer neurons [41,52–54]. Unlike in other fish species, most MCs in zebrafish innervate a single glomerulus within a distance of less than 40 μm from the soma [53] (Figure S1). Because the zebrafish OB is only about 500 μm in diameter, most neurons are optically accessible. The hierarchical chemotopic organization of glomerular activity maps has been studied previously [9,10]. TDCa imaging now permits the analysis of spatiotemporal odor response patterns across a large fraction of the OB neurons downstream of glomerular inputs.

In this study, we addressed two questions. First, we quantified basic odor response properties of INs, because this knowledge is important to understand how inhibitory interactions in the OB shape odor-encoding activity patterns across output neurons. The results show that IN responses exhibit a broader range of odor selectivities than MCs and develop differently over time during an odor response. Second, we directly analyzed the topological organization of MC and IN response patterns during the dynamic reorganization of OB output activity. Shortly after response onset, chemotopic activity maps were present throughout all layers and particularly pronounced across MCs. During the subsequent few hundred milliseconds, however, the chemotopy of MC activity patterns became substantially reduced, indicating that the chemotopic organization of odor representations is not maintained during pattern processing. Nevertheless, the transient chemotopy of MC activity patterns may be involved in computations that enhance pattern discriminability and thus may play an important role in circuit function.

## Results

### Measurements of Odor Responses from Populations of MCs and INs

**Figure 1:**
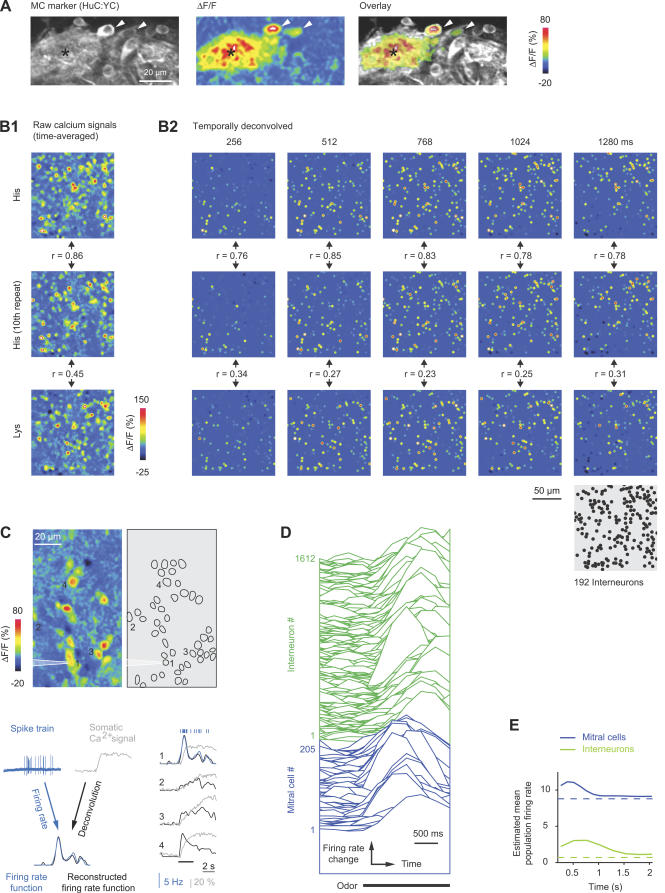
Temporally Deconvolved Ca^2+^ Imaging of Odor-Evoked Activity Patterns in the OB (A) Left: expression of the MC marker, HuC:YC, in the glomerular/MC layer. Center: changes in rhod-2 fluorescence evoked by odor stimulation (Tyr, 10 μM) in the same view. Right: overlay (thresholded). Arrowheads depict two responsive MC somata; asterisk depicts glomerular neuropil. (B) B1: Time-averaged raw Ca^2+^ signals from INs evoked by two applications of His (10 μM) and one application of Lys (10 μM). Between the first and second response to His, 17 other stimuli (His or Lys) were presented (unpublished data). *r,* correlation coefficient. B2: Activity maps in successive time windows after temporal deconvolution of Ca^2+^ signals (same trials as in B1). Each dot represents the position of one IN; colors represent the magnitude of the TDCa signal (color scale from −6 to 36; arbitrary units). Maps were low-pass spatially filtered to mimic the appearance of raw data. The position of each IN (*n* = 192 INs) is shown in the gray map (lower right). r, correlation coefficient. (C) Top left: spatial pattern of time-averaged Ca^2+^ signals evoked by odor stimulation (food extract) in the granule cell layer. Top right: locations of all somata in the field of view (*n* = 45). Action potentials from neuron 1 were recorded simultaneously in the loose-patch configuration. Bottom left: reconstruction of firing-rate changes from Ca^2+^ signals by temporal deconvolution, exemplified by response of neuron 1. Bottom right: reconstruction of firing-rate changes from somatic Ca^2+^ signals of four neurons. Action potentials and firing-rate function measured by electrophysiology are overlaid for neuron 1. Bar indicates odor presentation. (D) Temporally deconvolved Ca^2+^ signals of 205 MCs (blue) and 1,612 INs (green) in the same OB during odor stimulation (bar; Ala, 10 μM). Order of neurons is arbitrary. MC and IN responses are scaled differently. (E) Estimated mean population firing rates as a function of time. Dashed lines show spontaneous firing rates determined by electrophysiological recordings.

We measured neuronal activity patterns in the absence of anesthetics in an explant preparation of the entire zebrafish brain, including the nose and other sensory organs [32]. Neurons in the OB were loaded with the red-fluorescent Ca^2+^ indicator, rhod-2-AM, by bolus injection [46,47,51], and fluorescence was measured by two-photon microscopy [44,45]. MCs were identified by colocalization of rhod-2 fluorescence signals with a yellow-fluorescent MC marker protein (yellow cameleon [YC]) in a transgenic line (HuC:YC) [51,54] (Figure 1A). YC-negative neurons were collectively classified as INs. Because most recorded INs were located in the deep (granule cell) layer, we assume that IN datasets contained predominantly granule cells. Glomerular neuropil was identified as regions in the peripheral layer of the OB that were devoid of somata and contained a high density of MC dendrites (Figure 1A).

Application of odors to the nose caused robust changes in rhod-2 fluorescence in stimulus-specific subsets of neurons (Figure 1A and 1B1). Responses from up to approximately 350 individual neurons could be detected simultaneously in a given focal plane. Patterns of Ca^2+^ signals evoked by repeated stimulation with the same odor were reproducible and stable over time (Figures 1B and S2; average correlation coefficient [mean ± standard deviation (SD)]: MC patterns, 0.84 ± 0.03; IN patterns, 0.87 ± 0.03).

Somatic Ca^2+^ signals were low-pass filtered and temporally deconvolved using single-exponential kernels with appropriate time constants to convert Ca^2+^ signals into firing-rate changes (Figure 1C; see also Materials and Methods and [51]). The temporal resolution was limited by the frame rate (128 or 256 ms/frame) under our experimental conditions. This procedure yielded time series of frames, each representing the instantaneous firing rate of many neurons relative to the baseline firing rate (Figure 1B2). Individual MCs and INs responded to the same stimulus with different latencies and time courses (Figure 1B2, 1C, and 1D). As a consequence, the pattern of activity across the population changed over time. The sequence of patterns was reproducible upon repeated stimulation with the same odor, but distinct in response to different stimuli (Figure 1B2; see below).

In order to measure activity patterns across large, three-dimensional populations of neurons, odor stimuli (duration, ~2.4 s) were applied repeatedly, and Ca^2+^ signals were measured at different focal planes. We concentrated on the ventrolateral OB, which contains approximately 120 small glomeruli responding preferentially to amino acids [9,10,19]. Amino acids are important odors for most or all aquatic species [55] and thus represent a well-defined subspace of the natural odor space. The concentration used (10 μM) is in the intermediate physiological range [55] and does not saturate glomerular responses in zebrafish [9]. Three datasets were collected: dataset 1 contained responses from 1,313 MCs in *n* = 9 different OBs (mean ± SD: 146 ± 45 MCs per OB) to 16 amino acids (10 μM), acquired at 128 ms/frame. Dataset 2 contained responses of 5,111 INs (*n* = 3 OBs; 1,704 ± 161 INs per OB) to the same stimuli. Frame time was 256 ms/frame to allow for a larger field of view. Dataset 3 contained experiments in which responses to nine amino acids were measured from both MCs and INs in the same OBs (*n* = 3 OBs; mean ± SD: 265 ± 52 MCs and 1,388 ± 254 INs per OB) at 256 ms/frame (Figure 1C). The total number of neurons in the adult zebrafish OB is estimated to be about 20,000, including approximately 1,500 MCs [53,54]. Responses were therefore obtained from approximately 10% of all MCs or INs in each dataset. The fraction of amino acid–responsive neurons contained in our datasets was probably substantially higher because experiments were performed specifically in the amino acid–sensitive region.

### Basic Response Properties of MCs and INs

Although odor responses of MCs have been studied extensively in various species, response properties of INs are not well understood in vertebrates. We therefore compared basic response properties of MCs and INs. Peak times and rise times of excitatory responses were determined in all trials in which the TDCa signal exceeded a threshold well above noise level (TDCa signal ≥ 5; dataset 3; *n* = 2,218 MC responses and *n* = 21,754 IN responses; Materials and Methods). On average, MC responses became maximal 415 ± 327 ms (mean ± SD) after stimulus onset, whereas IN responses peaked significantly later (738 ± 492 ms; *p* < 0.001; Wilcoxon rank sum test). The mean rise time of the TDCa signal was also slightly, but significantly, longer for IN responses (MCs: 332 ± 214 ms; INs: 403 ± 333 ms; mean ± SD; *p* < 0.001, Wilcoxon rank sum test). In response to different odors, peak and rise times varied slightly, but were consistently lower for MC responses than for IN responses (unpublished data).

To estimate the mean population firing rates of MCs and INs, TDCa signals were averaged across all odors and neurons (datasets 1 and 2), scaled using factors derived from simultaneous electrophysiological recordings [51] to yield firing-rate changes, and offset corrected for spontaneous firing rates determined by electrophysiological recordings (see Materials and Methods). The estimated mean firing rate across the MC population increased during the initial phase of the odor response and decreased again thereafter (Figure 1E). The absolute firing-rate change was, however, small because individual responses could be excitatory or inhibitory, and because responses were sparse (see below). This observation is consistent with electrophysiological results [32]. The estimated population activity of INs increased more slowly, reached its peak slightly later, and was always substantially lower than that of MCs (Figure 1E).

**Figure 2:**
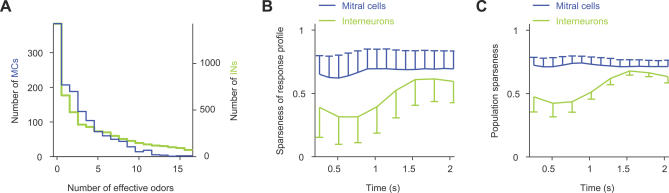
Selectivity and Density of Odor Responses (A) Histogram of response selectivity of MCs and INs for the 16 amino acid stimuli. (B) Mean sparseness (± SD) of MC and IN response profiles (“odor selectivity”) as a function of time. (C) Mean population sparseness (± SD) of MC and IN response patterns as a function of time.

The response selectivity of MCs and INs was quantified by two approaches. First, we determined how many of the 16 amino acids evoked a response for each neuron in datasets 1 and 2. A response was counted when the distribution of TDCa signals between 0 and 1.5 s after stimulus onset was significantly different from the distribution of TDCa signals during spontaneous activity (Kolmogorov-Smirnov test; *p* < 0.01). Using this criterion, MCs responded, on average, to 2.7 ± 3 of the 16 stimuli (mean ± SD), and very few MCs responded to more than ten stimuli. INs, in contrast, showed a broader range of odor selectivities (Figure 2A), and some INs responded to most or all stimuli. On average, INs responded to significantly more stimuli than MCs (4.1 ± 4.5; *p* < 0.001). Similar differences in response selectivity between MCs and INs were observed with a less stringent criterion for the detection of responses (Kolmogorov-Smirnov test; *p* < 0.05; unpublished data). Second, we quantified response selectivity as a function of time by the sparseness of excitatory response profiles (Figure 2B). This measure is one when a neuron responds with excitation to only one odor and zero when a neuron does not discriminate between stimuli [32,56]. The mean sparseness of MC response profiles was relatively high and almost constant throughout the odor response, consistent with electrophysiological results [32]. Sparseness of IN response profiles, in contrast, was lower, particularly during the early phase of a response. Hence, INs responded, on average, less selectively to odors than MCs.

To quantify the density of population activity, we calculated the sparseness of excitatory TDCa signals across the population of MCs or INs in successive time windows. This “population” sparseness is one when only one neuron is excited by a given stimulus and zero when all neurons respond equally. The mean population sparseness of MC activity was relatively high and almost constant throughout the odor response, consistent with electrophysiological observations [32]. The population sparseness of IN activity patterns, in contrast, was substantially lower and increased at later time points (Figure 2C; see also Figure 1B2). The density of the IN population response was therefore higher than that of the MC population response.

### Similarity Relationships between Activity Patterns

**Figure 3:**
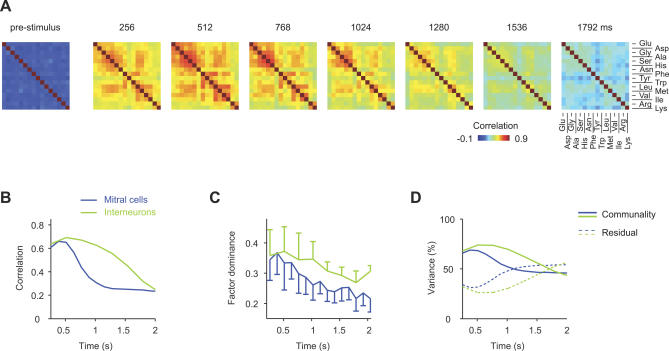
Similarity Relationships between Odor-Evoked Activity Patterns (A) Color-coded correlation matrices depicting the pairwise similarity between IN activity patterns evoked by different odors in successive 256-ms time windows (dataset 2). Left: correlations between activity patterns before stimulus onset. (B) Average correlation between initially similar activity patterns as a function of time. For each cell type and odor pair, maximal correlation coefficients were determined (datasets 1 and 2). The ten odor pairs yielding highest correlations were selected and their average correlation coefficients plotted as a function of time. (C) Mean factor dominance (± SD) as a function of time (datasets 1 and 2). Factor dominance quantifies the tightness of pattern grouping based on pattern similarity (Materials and Methods). (D) Mean percentage of variance explained by the four factors (“communality”; solid lines) as a function of time (datasets 1 and 2). Dashed lines show the fraction of variance that is not accounted for by factors (“Residual”).

MC activity patterns evoked by closely related stimuli in zebrafish are initially similar but become continuously more distinct during the first few hundred milliseconds of an odor response [32–34,51]. This pattern decorrelation may be involved in odor discrimination behaviors and other tasks [57–60]. The similarity relationships between IN activity patterns are, however, unknown. We therefore measured IN activity patterns by TDCa imaging and analyzed similarity relationships between IN activity patterns by correlation analysis and factor analysis. We first quantified the pairwise similarities between IN activity patterns evoked by the 16 odors (dataset 2) by their correlation coefficients in successive time windows (length, 256 ms). Results were plotted as a series of correlation matrices in which odors are arranged according to their presumed molecular similarity (Figure 3A). During the first few hundred milliseconds of the odor response, all correlation coefficients were positive, indicating that even dissimilar stimuli evoked partially overlapping IN activity patterns (e.g., His and Lys, Figure 1B2). However, highest correlation coefficients occurred near the diagonal of the correlation matrix, indicating that IN activity patterns evoked by related odors were most similar to each other. Subsequently, correlation coefficients decreased but remained elevated for a substantial period of time after stimulus onset (>1.7 s).

Highest correlations between IN activity patterns were evoked by stimulus pairs that also evoked high correlations between MC activity patterns during the initial phase of the odor response [32,33,51]. The temporal development of correlations was, however, different for MC and IN activity patterns. In order to compare the time course of correlation changes between MC and IN activity patterns, the average correlation coefficients for the ten pairs of stimuli whose activity patterns were most highly correlated during the initial phase of the odor response were plotted as a function of time (datasets 1 and 2; Figure 3B). Correlations between MC activity patterns decreased substantially during the first few hundred milliseconds to an intermediate level and remained stable thereafter. Correlations between IN activity patterns, in contrast, remained high during the decorrelation of MC activity patterns and slowly decreased only thereafter (Figure 3B).

Similarity relationships between activity patterns were further characterized by factor analysis [61,62]. This method extracts a small set of “elementary” patterns, called factors, from the original set of measured patterns. The association between each original pattern and each factor is quantified by “factor loadings.” When a dataset contains groups of similar patterns, the corresponding stimuli show high loadings on one common (“dominant”) factor, but low loadings on other factors. When the original patterns are dissimilar (decorrelated), factor dominance is low, i.e., each original pattern shows moderate loadings on multiple factors. Similarly, the fraction of variance explained by the factors (“communality”) is usually high when groups of patterns are similar, and lower otherwise. Factor dominance and communality are therefore indicators for “similarity groups” within a set of patterns. We extracted four factors from activity patterns evoked by the 16 amino acids across MCs and INs (datasets 1 and 2) in successive 256-ms time windows (Figure S3). For MC activity patterns, factor dominance was initially high but decreased during the subsequent few hundred milliseconds of the response (Figure 3C). For IN activity patterns, factor dominance was also maximal during the early phase of the response but decreased more slowly. Similar observations were made for the communality (Figure 3D). Factor analysis results therefore show that related IN activity patterns remain similar as MC activity patterns decorrelate and become only gradually more dissimilar at later times, consistent with results from correlation analysis.

### Topology of Odor-Evoked Activity Patterns

**Figure 4:**
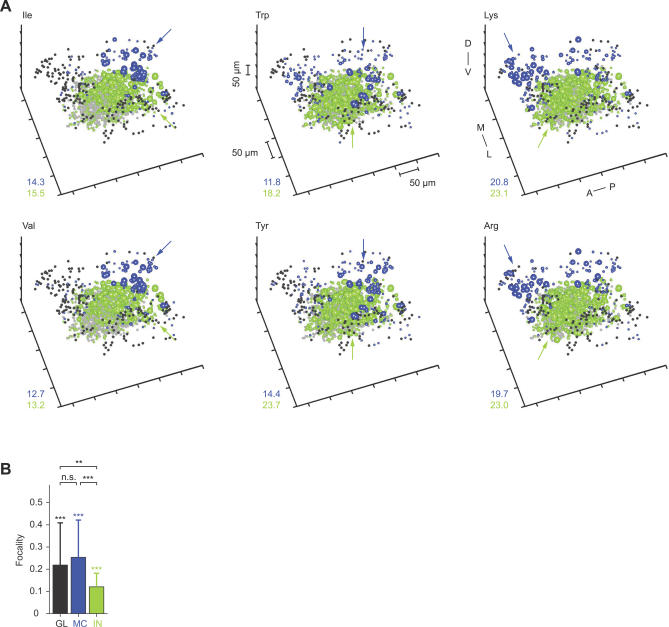
Three-Dimensional Reconstruction of Activity Patterns during the Initial Phase of the Odor Response (A) Three-dimensional patterns of TDCa signals evoked by six odors in one OB, time-averaged between 0 and 768 ms. Each plot symbol depicts the position of an individual neuron. Black dots and blue spheres represent MCs, gray dots and green spheres represent INs. Dots depict neurons with TDCa signals less than 10% of the maximum; colored spheres depict neurons with signals equal to or greater than 10% of the maximum. The size of spheres represents the magnitude of the TDCa signal between 10% and 90% of the maximum for each cell type and plot. Maxima were determined by averaging the largest 10% of TDCa signals and are reported in the lower left of each plot. Units are arbitrary; quantitative comparisons are valid between different activity patterns across the same cell type, but not between MCs and INs. Arrows indicate regions of dense activity (foci). Distance between ticks is 50 μm on all axes. Orientation is as indicated: anterior to posterior (A–P), medial to lateral (M–L), and dorsal to ventral (D–V). (B) Average focalities of odor-evoked activity patterns across glomeruli (GL), MCs (MC), and INs (IN). Glomerular activity patterns (raw Ca^2+^ signal; dataset 1) were time-averaged over 2.4 s; MC and IN activity patterns (TDCa signal; datasets 1 and 2) were averaged between 0 and 768 ms. Error bars show SD. Asterisks above each bar indicate statistically significant differences between measured focalities and the focalities of randomized activity patterns. Asterisks above brackets indicate statistically significant differences between patterns across glomeruli, MCs and INs. Double asterisks (**) indicate *p* ≤ 0.01; triple asterisks (***) indicate *p* < 0.001. n.s., not significant.

Response patterns across MCs and INs were reconstructed in three spatial dimensions to analyze their topological organization. We first concentrated on the initial phase of the odor response and analyzed TDCa signals time-averaged over the first 768 ms (shorter time windows yielded similar results). MC and IN activity patterns recorded in the same OB (one experiment from dataset 3) are shown in Figure 4A. MC activity was widespread but not uniformly distributed. Rather, activity patterns often contained volumes in which MC activity was particularly dense (Figure 4A, blue arrows). IN activity was more widespread but also exhibited volumes of dense activity that were usually associated with dense MC activity in the overlying MC layer (Figure 4A, green arrows). Hence, MC and IN activity patterns contained topologically related foci of activity.

The focality of activity patterns was quantified by an index that is zero when active neurons are randomly distributed and approaches one when active neurons are tightly clustered (Materials and Methods). The average focality of IN activity patterns was lower than that of MC activity patterns (Figure 4B). To test whether the focalities were significantly different from randomly distributed activity, the positions of neurons in measured patterns were randomly permuted. The focality of randomized patterns was always near zero and significantly different from that of activity patterns across MCs or INs. The gross distribution of activity across MCs and INs during the initial phase of the odor response is therefore odor-dependent and topologically related across layers.

We also estimated the focality of glomerular activity patterns from Ca^2+^ signals measured in glomerular neuropil regions (Figure 1A) in the same OBs as MC activity patterns (dataset 1). Ca^2+^ signals were time-averaged over 2.4 s and not temporally deconvolved, because transmitter release from olfactory sensory neurons is nearly linearly related to the intracellular Ca^2+^ concentration [63], and because responses of sensory neurons in zebrafish do not change much over time [32,34]. The focality of glomerular activity patterns was not significantly different from that of MC activity patterns but higher than that of IN activity patterns (Figure 4B). Because Ca^2+^ signals from glomerular neuropil may not exclusively reflect the activity of sensory inputs, we also quantified the focality of glomerular Ca^2+^ signals evoked by the same set of stimuli after selectively loading a dextran-coupled Ca^2+^ indicator into sensory neurons (data from ref. [9]; measured using wide-field optics). Again, the focality of glomerular activity patterns was not significantly different from the focality of MC activity patterns (*p* = 0.38) but significantly higher than the focality of IN activity patterns (*p* < 0.001). These results suggest that the focality of MC activity patterns during the initial phase of the odor response reflects, at least in part, the focality of glomerular inputs.

Biochemical and physiological studies in different fish species indicate that groups of amino acids sharing certain chemical features stimulate overlapping, albeit not identical, sets of odorant receptors and olfactory receptor neurons (ORNs), whereas amino acids with different chemical features stimulate largely non-overlapping sets of receptors and ORNs [9,64,65]. Based on these studies, amino acids can be assigned to chemical groups termed “long-chain” (e.g., Ile and Val), “aromatic” (e.g., Trp, Tyr, and Phe), and “basic” (e.g., Lys and Arg), after the feature that seems to influence receptor binding. Stimuli of the same chemical group tend to activate common glomeruli within a defined region so that the associated chemical features are represented in a chemotopic fashion across the array of glomeruli [9]. We therefore examined whether stimuli of these chemical groups evoke overlapping and localized response patterns also in neurons downstream of glomeruli.

MC activity patterns evoked by stimuli of the same chemical group (e.g., Ile and Val, Trp and Tyr, and Lys and Arg) overlapped substantially and had a focus in the same region (Figure 4A). Moreover, foci of activity evoked by stimuli from different chemical groups were spatially separated from each other. Foci of activity evoked by long-chain, aromatic, and basic amino acids were consistently found in a posterior, central, and anterior location, respectively (Figure 4A), in different animals. These locations correspond to the location of foci in glomerular activity patterns associated with the same chemical features [9]. IN activity evoked by stimuli of different chemical groups was generally more widespread but was also densest in regions corresponding to foci of glomerular or MC activity. Hence, the rough chemotopic map observed at the level of glomerular inputs appears to be reflected in MC activity patterns and, to a lesser extent, in IN activity patterns shortly after response onset.

### Topological Reorganization of Activity Patterns

**Figure 5:**
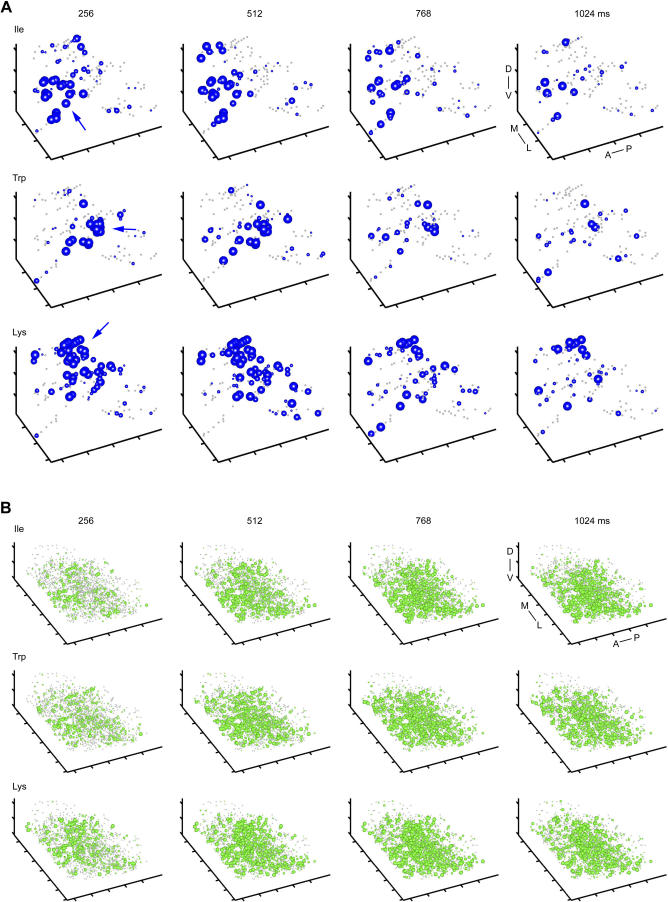
Topological Reorganization of Activity Patterns (A) Three-dimensional MC activity patterns evoked by three odors in successive 256-ms time bins (*n* = 131 MCs). Sphere size represents TDCa signal amplitude between 1 and 5 (arbitrary units); dots depict MCs with TDCa signals less than 1. Arrows indicate foci of MC activity during early time bins. Distance between ticks is 50 μm on all axes. Orientation is as indicated: anterior to posterior (A–P), medial to lateral (M–L), and dorsal to ventral (D–V). (B) Three-dimensional IN activity patterns evoked by the same odors in successive 256-ms time bins (*n* = 1,612 INs). Sphere size depicts TDCa signal between 2 and 10 (arbitrary units); dots depict INs with TDCa signals less than two. Distance between ticks is 50 μm on all axes.

We next analyzed three-dimensional activity patterns as time series in successive 256-ms time windows to examine how the topological organization of activity patterns changes during the reorganization of OB output activity. Shortly after response onset, MC activity patterns contained foci of activity at locations typical for the chemical feature of the stimulus (Figure 5A, blue arrows), consistent with the observations in activity patterns time-averaged over the first 768 ms (Figure 4A). During the subsequent few hundred milliseconds, however, foci became less pronounced and activity became more evenly distributed (Figure 5A; Videos S1–S3). IN activity patterns also changed over time, but broad foci were still observed a few hundred milliseconds after response onset (Figure 5B; Videos S1–S3).

**Figure 6:**
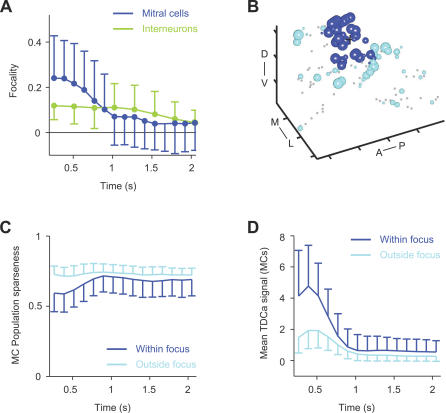
Focality of Activity Patterns and Local Sparsening (A) Mean focality (± SD) of MC and IN activity patterns as a function of time (datasets 1 and 2). Dots indicate significant differences (*p* < 0.05) from the focality of randomized patterns. (B) Assignment of MCs to the focus illustrated by three-dimensional activity pattern 256 ms after response onset (Lys, same as in Figure 4A; same conventions). Black cross depicts location of centroid. MCs within 50 μm from the centroid are classified as within the focus (blue); the remaining MCs are classified as outside the focus (cyan). Distance between ticks is 50 μm. Orientation is as indicated: anterior to posterior (A–P), medial to lateral (M–L), and dorsal to ventral (D–V). (C) Population sparseness of MC activity within foci (blue) and outside foci (cyan) as a function of time, averaged over all odors and OBs (± SD; dataset 1). (D) Mean TDCa signal (representing firing-rate change; arbitrary units) of MC activity within foci (blue) and outside foci (cyan) as a function of time, averaged over all odors and OBs (± SD; dataset 1).

We then quantified the focality of MC and IN activity patterns in successive time bins. The average focality of MC activity patterns decreased significantly during the first few hundred milliseconds (*r* = −0.92, *p* < 0.001). The decrease in focality was particularly pronounced for those patterns that were initially highly focal (Figure S4A). The focality of IN activity patterns was lower initially, remained almost constant during the subsequent phase of the odor response, and decreased only slightly at late time points (Figure 6A; *r* = −0.93; *p* > 0.001). Focalities did, however, remain significantly different from the focality of randomized patterns. Different focality indices yielded similar results (Figure S4B and S4C). OB output activity is therefore topologically reorganized and becomes more uniformly distributed during the first few hundred milliseconds of an odor response.

Odor-evoked MC activity may become more evenly distributed because activity is sparsened within foci, or because it becomes denser outside the foci. To distinguish between these possibilities, we quantified population sparseness separately within and outside the volumes corresponding to foci. For each activity pattern, the centroid (“center of mass,” where the mass is representing the TDCa signal) was determined 256 ms after response onset. For each stimulus, MCs within a 50-μm radius from the centroid were classified as belonging to the focal volume, whereas MCs more than 50 μm away from the centroid were classified as outside the focal volume (Figure 6B). The average sparseness of activity within the focal volume was initially lower than that outside the focal volume (Figure 6C), reflecting the high density of active MCs in the focus. During the first few hundred milliseconds, however, sparseness in the focal volume increased markedly and approached the sparseness outside the foci. Outside the focal volume, the sparseness remained almost constant. Consistent with these observations, the average TDCa signal of neurons within foci was initially higher than outside foci but then decreased substantially (Figure 6D). Hence, OB output activity becomes more evenly distributed because MC activity patterns are locally sparsened within the initial foci. This result is consistent with the visual inspection of three-dimensional MC activity patterns in successive time bins (Figure 5A).

**Figure 7:**
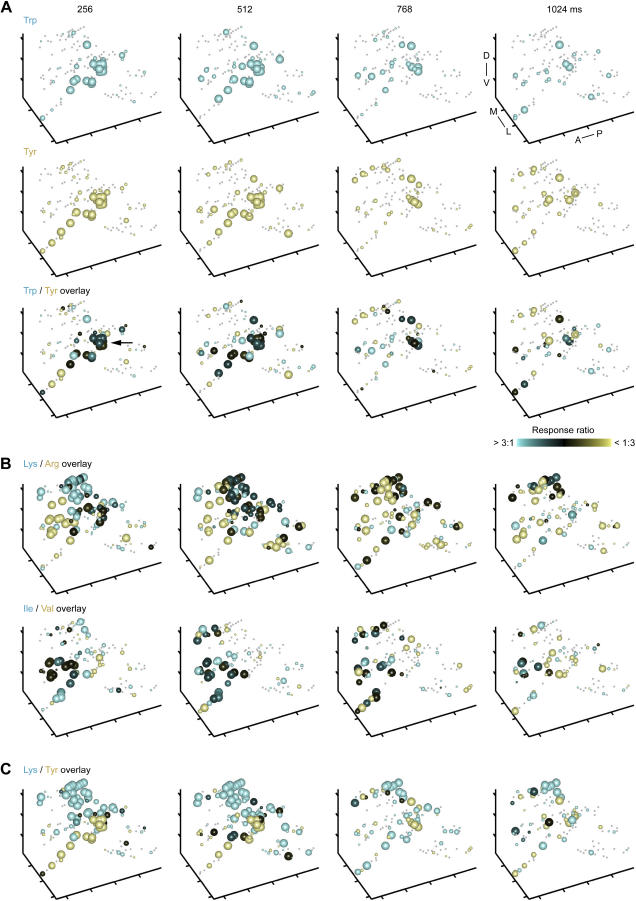
Reduction of Overlap between MC Activity Patterns over Time (A) Top and middle: Three-dimensional MC activity patterns evoked by two related stimuli (Trp and Tyr) in successive 256-ms time bins (same as in Figure 5A; same conventions; *n* = 131 MCs). Bottom: overlay. The size of spheres represents the magnitude of the larger response to the two stimuli; the color indicates the response ratio. Pure colors (yellow or cyan) indicate that a given MC responded predominantly to one of the two stimuli; black indicates similar responses to both stimuli. Arrow indicates high degree of overlap in focus during the initial phase of the response. Distance between ticks is 50 μm on all axes. Orientation is as indicated: anterior to posterior (A–P), medial to lateral (M–L), and dorsal to ventral (D–V). (B) Further examples of MC response patterns evoked by chemically related stimuli (overlays; same conventions as in [A]). (C) Overlay of MC response patterns evoked by two dissimilar stimuli. Same conventions as in (A).

The overlap of MC activity within foci is likely to contribute significantly to the initially high correlation between MC activity patterns evoked by chemically similar stimuli. We therefore examined whether the local sparsening of MC activity patterns contributes to pattern decorrelation during the first few hundred milliseconds. Local sparsening in the focus would promote decorrelation if it is odor-specific, i.e., if different MCs continue to be active in response to chemically similar stimuli. To address this question, we overlaid time series of three-dimensional activity patterns evoked by chemically related stimuli. As shown in Figure 7A, the overlap of MC activity patterns evoked by stimuli from the same chemical group (Trp and Tyr; aromatic) was initially highest in the focus (black spheres in overlay; arrow) and decreased as activity patterns were locally sparsened. Similar observations were made when activity patterns evoked by stimuli from other chemical groups were compared (Figure 7B; “long-chain”: Ile and Val; and “basic”: Arg and Lys). Activity patterns evoked by stimuli from different chemical groups (e.g., Lys and Tyr; Figure 7C) showed little overlap throughout the odor response. Hence, local sparsening of MC activity patterns within foci is stimulus-specific and contributes to the decorrelation of OB output activity patterns.

**Figure 8:**
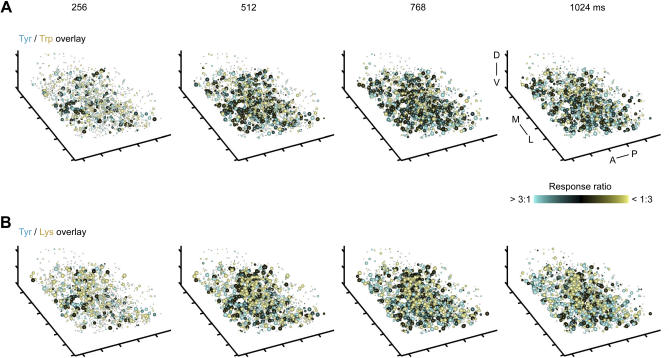
Comparison of Three-Dimensional IN Activity Patterns (A) Overlay of IN response patterns evoked by chemically related odors (Trp and Tyr) in successive 256-ms time windows (same as in Figure 5B; *n* = 1,612 INs). Conventions as in Figure 7A. Sphere size depicts TDCa signal between 2 and 10 (arbitrary units); dots depict INs with TDCa signals less than 2. Distance between ticks is 50 μm on all axes. Orientation is as indicated: anterior to posterior (A–P), medial to lateral (M–L), and dorsal to ventral (D–V). (B) Overlay of IN response patterns evoked by two dissimilar stimuli. Same conventions as in (A).

Overlays of IN activity patterns evoked by chemically similar stimuli (e.g., Lys and Arg) showed little or no reduction in the overlap during the initial phase of the odor response (Figure 8A), consistent with the observation that correlations between IN activity patterns remain high during the decorrelation of MC activity patterns (Figure 3A and 3B). The overlap between IN activity patterns evoked by dissimilar stimuli (e.g., Lys and Tyr) was lower but still substantial (Figure 8B). These observations are consistent with results from correlation analysis (Figure 3A).

### Chemotopy of Odor-Evoked Activity Patterns

**Figure 9:**
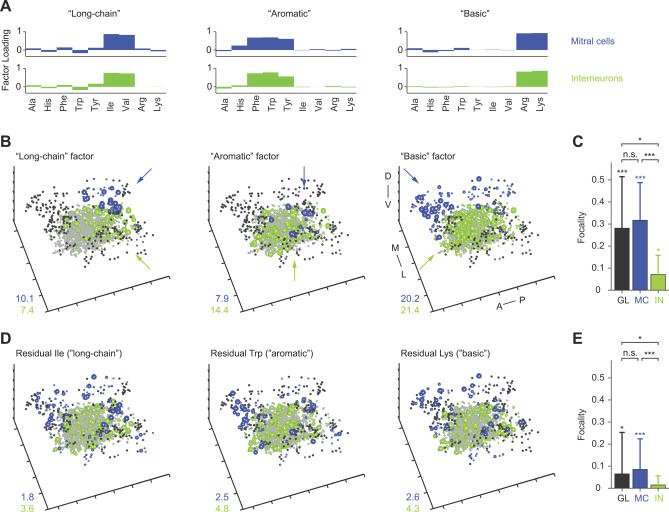
Chemotopy of MC and IN Activity Patterns during the Initial Response Phase (A) Distribution of factor loadings for three of four factors extracted from MC and IN activity patterns evoked by nine stimuli in one OB (time-averaged between 0 and 768 ms; same experiment as in Figure 4A). Each factor is associated with a group of chemically related stimuli and represents a distinct chemical feature (“long-chain,” “aromatic,” and “basic”). (B) Three-dimensional reconstructions of the three factors representing long-chain, aromatic, and basic properties. Same experiment and conventions as in Figure 4A. Distance between ticks is 50 μm. Arrows depict regions of dense activity. Orientation is as indicated: anterior to posterior (A–P), medial to lateral (M–L), and dorsal to ventral (D–V). (C) Average focalities of factors extracted from activity patterns across glomeruli (GL), MCs (MC), and INs (IN). Glomerular activity patterns (raw Ca^2+^ signal; dataset 1) were time-averaged over 2.4 s; MC and IN activity patterns (TDCa signals) were averaged between 0 and 768 ms (datasets 1 and 2). Conventions as in Figure 4B. A single asterisk (*) indicates *p* ≤ 0.05; triple asterisks (***) indicate *p* < 0.001. n.s., not significant. (D) Three-dimensional reconstructions of pattern components that are not explained by factors (Residual) for three stimuli associated with different factors. Same experiment and conventions as in (B) and Figure 4A. (E) Average focalities of residual patterns (time-averaged between 0 and 768 ms; datasets 1 and 2). Conventions as in (C) and Figure 4B.

In a chemotopic map, different molecular features are associated with neural activity in different regions. The visual inspection of activity patterns revealed that stimuli of different chemical groups evoke foci of activity in distinct regions during the early phase of an odor response (Figures 4A, 5A, and 7), suggesting the presence of a transient chemotopic map. In order to directly address this question, we tested whether neurons representing a molecular feature are indeed spatially clustered, using factor analysis. A factor is a hypothetical activity pattern that represents the “elementary” molecular feature shared by a set of stimuli associated with the factor [61,62]. The spatial distribution of activity within factors therefore directly reflects the topology of feature representations. Moreover, the focality of factors provides a quantitative measure of chemotopy. We first concentrated on the initial phase of the odor response and analyzed MC and IN response patterns time-averaged over the first 768 ms (shorter time windows yielded similar results). In addition, we extracted factors from glomerular activity patterns, estimated as described above. Four factors were extracted from response patterns to the 16 odors in datasets 1 and 2 and to the nine odors in dataset 3. The association between stimuli and factors was very similar for glomerular, IN, and MC activity patterns: in all OBs except for one OB in dataset 1, three of the four factors were consistently associated with the chemical groups of stimuli defined previously (“basic,” “aromatic,” and “long–chain”; Figures 9A and S3). The fourth factor was associated with variable groups of odors in different animals and therefore was excluded from further analysis.

Three-dimensional reconstructions of the extracted factors had clear foci of activity (Figure 9B, arrows). The focalities of factors extracted from glomerular and MC activity patterns were not significantly different, whereas the focality of IN activity patterns was lower (Figure 9C). However, the average focality of factors extracted from all datasets was significantly different from the focality of randomized patterns (Figure 9C). Foci of activity in IN factors were associated with foci in the corresponding MC factors in more superficial layers (Figure 9B). Moreover, foci of activity in factors corresponded to foci in patterns evoked by stimuli associated with each factor (compare Figures 9B and 4A). Hence, activity patterns across MCs and INs contain topologically related chemotopic maps during the early phase of an odor response.

The extracted factors accounted only for approximately 50%–70% of the variance in the measured activity patterns (Figure 3D). Each individual activity pattern therefore also had a unique component. These unique components (“residuals”) contain not only trial-to-trial variability, but also information about the precise identity of a given odor. We therefore reconstructed residuals in three dimensions and found that they were substantially more widespread than factors (Figure 9D and 9E). Active neurons in residuals were found within the volumes corresponding to foci in factors as well as in the surrounding areas. Thus, information about precise odor identity appears to be widely distributed.

Previous low-resolution imaging of sensory input to glomeruli revealed foci of activity representing “basic,” “aromatic,” and “long-chain” features also in patterns of glomerular input activity in zebrafish [9]. These foci are segregated along the anterior–posterior axis and preserved across individuals. We therefore examined whether chemotopic foci of MC and IN activity during the initial phase of the odor response are preserved between individuals and related to glomerular input activity. For each OB, the coordinates of glomeruli, MCs, and INs were centered on the centroid of the activity in the corresponding aromatic factor. Activity in the three factors was then binned in 40 × 40 × 40-μm^3^ voxels and averaged over individuals. When the geometric arrangement of chemotopic foci is consistent in different individuals, the distribution of activity in the averaged factors should be focal; otherwise, activity should be uniformly distributed.

**Figure 10:**
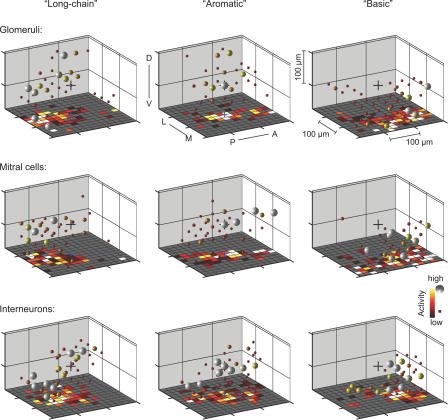
Stereotyped Chemotopy during the Initial Phase of the Odor Response Three-dimensional plots of factors representing distinct molecular features (long-chain, aromatic, and basic), averaged over individuals, during the early phase of the odor response. Factors were extracted from glomerular activity patterns (raw Ca^2+^ signals time-averaged between 0 and 2 s; dataset 1) and from MC and IN activity patterns (TDCa signals time-averaged between 0 and 768 ms; datasets 1 and 2) in individual OBs. Activity of factors was then binned in 40 × 40 × 40-μm^3^ voxels, centered on the centroid of the aromatic factor, and averaged over animals. Spheres represent voxels; color and size of spheres indicate average activity in each voxel. Voxels with activity smaller than an arbitrary threshold are not shown. Black cross indicates the centroid of the aromatic factor. Floor patterns are projections of three-dimensional activity distributions, binned in 20 × 20 × 20-μm^3^ voxels, onto the horizontal plane. Orientation is as indicated: posterior to anterior (P–A), lateral to medial (L–M), and dorsal to ventral (D–V). Blue cross indicates the centroid of the aromatic factor in the horizontal plane. Distinct volumes of dense activity in different factors are segregated along the anterior–ventral to posterior–dorsal axis. The topological organization of averaged factors is similar for glomeruli, MCs and INs, showing that chemotopic maps during the early phase of an odor response are topologically related across layers and stereotyped between animals.

Figure 10 shows three-dimensional plots of averaged factors. For easier inspection, two-dimensional projections into a horizontal plane are shown separately in the floor patterns of three-dimensional plots (Figure 10) and in Figure S5. In all averaged factors, activity was concentrated within a circumscribed volume. Foci in different factors were segregated in an anterior-to-posterior direction and partially along the ventral-to-dorsal axis also, consistent with glomerular activation patterns recorded previously [9]. The geometrical arrangement of foci representing long-chain, aromatic, and basic features was consistent in factors extracted from glomeruli, MCs, and INs. Hence, chemotopic maps during the initial phase of an odor response are at least partially preserved between individuals and topologically related across layers of the OB.

**Figure 11:**
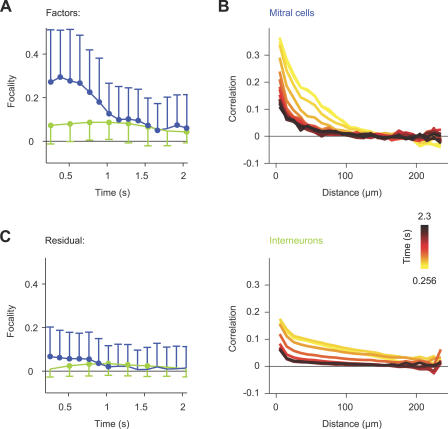
Topological Reorganization of Odor-Evoked Activity Patterns (A) Mean focality (± SD) of factors extracted from MC and IN activity patterns as a function of time (datasets 1 and 2). Dots indicate significant differences (*p* < 0.05) from the focality of randomized patterns. (B) Top: correlation between odor response profiles of MCs, averaged over all pairs of MCs, as a function of distance (10-μm bins; dataset 1). Color of curves indicates time after response onset. Bottom: same for odor response profiles of INs (dataset 2). (C) Average focality (± SD) of pattern components not accounted for by factors (Residual) as a function of time (datasets 1 and 2). Dots indicate significant differences (*p* < 0.05) from the focality of randomized patterns.

We then examined how chemotopy is affected by the local sparsening and topological reorganization of activity patterns during the initial phase of the odor response. Factors were extracted from MC and IN activity patterns in successive 256-ms time windows, and chemotopy was quantified by the focality of factors. The focality of factors extracted from IN activity patterns remained relatively stable. The focality of MC factors, in contrast, was initially high but then decreased during the first few hundred milliseconds after response onset (Figure 11A), similar to the focality of MC responses to individual odors (Figure 6A). The focality of factors extracted from both MCs and INs did, however, remain significantly different from the focality of randomized patterns (Figure 11A), indicating that chemotopy did not vanish completely. Similar results were obtained using different focality indices (Figure S4D and S4E). These data show that the chemotopy of OB output activity is substantially reduced during the topological reorganization of activity patterns in the OB.

To confirm these results by an independent analysis, we quantified the similarity of response profiles of individual neurons as a function of their distance. In chemotopically organized patterns, response profiles of nearby neurons are expected to be, on average, more similar than response profiles of distant neurons. Shortly after response onset, the mean correlation of response profiles was highest for nearby neurons and decreased with distance (Figure 11B, yellow curve). During the subsequent reorganization of activity patterns, response profiles of nearby neurons became less similar and the decrease in response profile similarity with distance became less pronounced (Figure 11B, orange to black curves). The distance dependence of the average correlation between IN response profiles was not as pronounced as for MCs, and the decrease in response profile similarity occurred later during the odor response (Figure 11B). These data confirm that the chemotopy of OB output activity patterns decreases during the topological reorganization of activity patterns.

As chemotopic maps disappeared, the fraction of the variance contained in unique pattern components (residuals) increased (Figure 3D, dashed lines) and OB output activity becomes more informative about the precise identity of odors [32–34]. One possible mechanism underlying this effect is that after odor-specific local sparsening, unique MC activity patterns emerge within areas that previously contained foci. In this scenario, residuals would be expected to become more focal over time because unique components would be increasingly dominated by neurons within the initial foci. Alternatively, the increased uniqueness of MC activity patterns may be caused by an increased contribution of odor-specific firing patterns outside foci because MCs within foci are generally inhibited. In this scenario, residuals should also become more focal because they would be dominated by activity outside initial foci. Both mechanisms could, however, also coexist. In this case, the focality of residuals should approach zero because odor-specific information would be conveyed by widely distributed MCs. For both MC and IN activity patterns, the average focality of residuals was low throughout the odor response. The focality of MC residual patterns approached zero and became indistinguishable from that of randomized patterns (Figure 11C). Hence, information about precise odor identity appears to be conveyed by distributed sets of neurons both within and outside areas of initial foci. This result is consistent with the visual observation that MCs responding specifically to one of two similar stimuli are widely distributed after local sparsening (Figure 7A and 7B).

In summary, the decorrelation of MC activity patterns (Figure 3B), the reduction in overlap between related MC activity patterns (Figure 7A and 7B), the decrease in factor dominance (Figure 3C), and the decrease in the communality of extracted factors (Figure 3D) indicate that MC firing-rate patterns become less informative about the molecular features that were initially represented in spatial maps. Factor analysis and the distance dependence of response profile similarity directly demonstrate that chemotopy of OB output activity patterns becomes substantially reduced during the first few hundred milliseconds of an odor response. The initial chemotopic mapping of chemical features is therefore not maintained during the topological reorganization of OB output activity.

## Discussion

Using TDCa imaging in the zebrafish OB, we measured spatiotemporal activity patterns evoked by a substantial fraction of the naturally occurring amino acids across a substantial fraction of the neurons involved in processing these stimuli. Previous results demonstrated that TDCa imaging is reliable and captures a large fraction of the variance in the original firing-rate changes [51]. The datasets obtained by TDCa imaging therefore enabled exhaustive and quantitative analyses of population activity patterns. We first compared basic response properties of INs to those of MCs and found that IN responses are less selective and slightly slower than MC responses. Moreover, activity patterns across the population of INs were initially more widespread and, on average, more highly correlated than MC response patterns. We then analyzed the topology of odor-evoked activity patterns to examine how chemotopically organized odor representations are processed in the OB. Whereas IN activity patterns exhibited low (but significant) chemotopy throughout an odor response, chemotopy of MC activity patterns was pronounced shortly after response onset. During the subsequent few hundred milliseconds, however, MC activity patterns were locally sparsened within regions of initial foci and topologically reorganized. As a consequence, chemotopy decreased, MC activity became more evenly distributed, and patterns became more informative about odor identity. The initial chemotopy of odor representations in the OB is therefore not maintained during pattern processing. Nevertheless, our results suggest that the transient chemotopic organization of MC activity plays a functional role in the optimization of OB outputs.

### Response Properties of Interneurons in the OB

In insects, INs were found to respond less selectively to odorants than projection neurons [66,67]. In vertebrates, biophysical properties of INs have been characterized in vitro [e.g., 26,68–70], and synaptic input and action potential firing of individual INs during odor responses have been examined in vivo [71–75]. Odor-evoked activity in populations of INs has been visualized at low resolution by 2-deoxyglucose uptake or immediate early gene expression [6,11,38–40]. To our knowledge, however, response profiles of individual INs to defined panels of relevant odors have not been studied systematically before.

Although MCs usually responded with excitation only to a few amino acids, a subset of INs was excited by most or all stimuli. This broad tuning may result from the convergence of MCs with different tuning profiles onto individual INs, from nonspecific centrifugal inputs, or both. Other INs, however, responded more selectively to odors. The population of INs therefore exhibited a wider range of odor selectivities than the population of MCs. Conceivably, INs with different tuning properties could subserve different functional roles. For example, broadly tuned INs may mediate general operations, such as synchronization or global gain control, whereas more selective INs may shape OB output patterns in an odor-dependent manner. It will now be interesting to determine whether INs with different response selectivities indeed subserve different functions, and whether they correspond to different types of neurons, such as periglomerular and granule cells.

Activity patterns across the population of INs were less sparse than MC activity patterns, particularly during the initial phase of the odor response. The spatial distribution of responding INs was not uniform, but more widespread than MC activity shortly after response onset. This may be the consequence of the divergent, yet spatially restricted, connectivity between MCs and INs. IN response patterns evoked by chemically related stimuli were more similar to each other than response patterns evoked by structurally different stimuli, as observed in MCs [32,51]. Nevertheless, the similarity relationships between IN activity patterns differed from those of MC activity patterns in at least two ways. First, IN response patterns were positively correlated even when odors were structurally dissimilar. MC response patterns evoked by dissimilar stimuli are, in contrast, nearly uncorrelated during the initial phase of the odor response. Second, correlations between IN activity patterns remain high during the first few hundred milliseconds, when MC activity patterns are decorrelated, and gradually decrease only at later time points. As MC activity patterns become decorrelated, INs therefore provide distributed, yet somewhat focal, patterns of inhibitory feedback that is related, but not identical, for similar stimuli. This topologically organized feedback may ensure dense inhibitory interactions between MCs receiving similar inputs.

Our results also demonstrate that IN activity patterns change during the course of an odor response in a stimulus-specific manner, consistent with the observation in brain slices that individual granule cells respond to brief electrical stimulation in superficial layers with reproducible, glomerulus-dependent latencies of up to approximately 900 ms [76]. Hence, the pattern of IN activity changes in a stimulus-dependent fashion in response to sensory input, although the pattern changes are qualitatively different from those observed in MCs. The resulting dynamics of inhibitory feedback onto the MC population is likely to contribute to the temporal patterning of OB output activity.

### Topological Reorganization of Odor-Evoked Activity Patterns in the OB

MC activity patterns were focal shortly after response onset but became more evenly distributed during the subsequent few hundred milliseconds. Hence, activity patterns transmitted from the OB to higher brain regions are topologically reorganized during pattern processing. This topological reorganization of OB output activity was associated with a disappearance of chemotopic maps. The chemotopic organization of glomerular activity patterns is therefore only transiently reflected at the next stage of the olfactory pathway.

The relationships between activity patterns across different populations of neurons provide initial insights into the mechanisms underlying pattern reorganization. During the early phase of an odor response, MC and IN activity patterns were topologically related to glomerular activity maps, suggesting that MC and IN activity patterns are initially driven to a large extent by glomerular input. The subsequent reorganization of activity patterns, however, implies that the chemotopic organization is unstable. This may be expected because inhibitory feedback from INs modifies MC activity, which in turn changes IN activity patterns. Because patterns of inhibitory feedback evoked by different stimuli are not identical, the pattern of inhibitory feedback onto MCs would modify OB output activity in an odor-specific manner. The reorganization of activity patterns is therefore likely to result, at least in part, from interactions between MCs and INs. After a few hundred milliseconds, OB output activity patterns stabilize [34], implying that feedback interactions between the populations of MCs and INs approach a dynamic equilibrium.

An important mechanism contributing to the topological reorganization of MC activity patterns is the local sparsening of MC activity within initial foci. Foci in the MC layer were associated with dense IN activity in deeper layers, suggesting that local sparsening of MC activity patterns is caused by topologically related inhibitory feedback from INs. One prediction from this hypothesis is that the focality of IN activity should be lower than the initial focality of MC activity because connections from MCs to INs are divergent. This was indeed observed. A second prediction is that inhibitory feedback should reduce, but not completely abolish, the focality of MC activity patterns to maintain a dynamic equilibrium. Indeed, the focality of MC and IN activity patterns remained significantly different from that of randomized patterns (Figures 6A and 11A). A third prediction is that MC activity should not spread from the focus to adjacent regions because, unlike in the insect antennal lobe [67,77], extensive interglomerular excitatory interactions between principal neurons have not been described in the vertebrate OB (but see [78,79]). Consistent with this hypothesis, the density of activity outside foci was stable during the odor response (Figure 6C). Finally, the reduction of chemotopy by topologically related inhibitory feedback and local sparsening could be reproduced in simulations (unpublished data). Hence, the topological reorganization of MC activity can be explained, at least in part, by local sparsening through divergent, yet topologically related, inhibitory feedback from INs. The general architecture of the OB and the hierarchical chemotopy of glomerular activation patterns are conserved in different vertebrate classes, suggesting that a similar topological reorganization of OB output patterns may also occur in other vertebrate species.

### Topological Reorganization of Activity Patterns and Neuronal Computations

How may the transient chemotopic organization of activity patterns influence the function of neuronal circuits in the OB? In parallel with the topological reorganization of activity patterns, initially similar MC activity patterns become decorrelated [32–34,51]. This computation increases the discriminability of odor representations and may be involved in odor discrimination behavior [57–59]. In theory, the topological reorganization and the decorrelation of MC activity patterns could occur independently. For example, chemotopy could be maintained during pattern decorrelation if activity was redistributed locally within foci. Likewise, correlations could be maintained despite a topological reorganization if all patterns were reorganized in the same manner. Alternatively, the topological reorganization of activity may be functionally involved in pattern decorrelation. In this case, the two processes would depend on each other and cannot be separated.

Detailed comparisons of three-dimensional activity patterns revealed that the overlap between MC responses to related stimuli is initially high within foci. Subsequently pattern overlap was decreased by odor-specific local sparsening, presumably because INs inhibit different subsets of MCs in response to each stimulus. Hence, local sparsening contributes both to the topological reorganization and to the decorrelation of MC activity patterns. This conclusion is further supported by factor analysis results. Using this technique, we extracted the pattern components that accounted for the high initial similarity of related patterns (factors), as well as the components that were unique to each pattern (residuals). The initial focality of factors demonstrated that pattern overlap was most pronounced in those regions where MC activity was dense, indicating that activity within foci dominates the initial correlation of related patterns. Unique components of activity patterns containing stimulus-specific information were, in contrast, widely distributed. Together, these results indicate that local sparsening contributes to pattern decorrelation in two ways: first, local sparsening decreases pattern overlap within regions of initially dense activity. Second, local sparsening reduces the overall activity within regions of greatest overlap so that the contribution of more-specific pattern components becomes enhanced. Local sparsening therefore promotes both the decorrelation and the topological reorganization of OB output patterns, indicating that the two phenomena are linked. One function of the transient chemotopy in the OB may therefore be to enable local sparsening and thereby enhance the decorrelation of related odor representations. Further experiments are required to explore this hypothesis.

The topological reorganization of activity patterns occurred less than 1 s after response onset. Individual components of activity patterns may, however, be reorganized more rapidly. In electrophysiological recordings, complete pattern decorrelation is observed approximately 800 ms after response onset when activity patterns are analyzed across all recorded MCs [32]. However, decorrelation specifically occurs within odor-specific components of the overall firing-rate patterns that are defined by the absence of phase locking to the local field potential oscillation. More specific analyses of these components revealed that pattern decorrelation is nearly complete already after 400 ms [33]. The time window during which a topological reorganization of the overall activity patterns was observed should therefore be considered as an upper time limit for the processing of specific pattern components.

We concentrated our analysis on the ventrolateral region of the zebrafish OB where secondary features of amino acids are mapped in “fine” chemotopic patterns. In addition, the OB of zebrafish and other vertebrates also exhibits a “coarser” chemotopic organization in which primary chemical features (“chemical classes”) are mapped to large, distant areas [11,12,17,21,80]. In zebrafish, for example, amino acids and bile acids activate glomeruli predominantly in the ventrolateral and dorsomedial OB, respectively [10]. This “coarse” chemotopic organization may not be affected by the topological reorganization observed here because the underlying sparsening of MC activity patterns is more local. It will now be interesting to examine whether “coarse” chemotopy is maintained and enables the parallel processing of distinct odor classes.

Within the amino acid–sensitive region, the chemotopic organization of OB output patterns is transient, raising the question as to what the function of fine chemotopic maps may be for odor processing. One possibility is that downstream neurons use the transient chemotopic map in the OB as a coordinate system to access sensory information about distinct chemical features during the initial phase of an odor response. Alternatively, the transient chemotopy may organize synaptic interactions within the OB. In general, a topographic feature map does not, per se, encode information, because the information conveyed by an activity pattern is independent of the neurons' positions. In a neuronal circuit, however, connections are usually formed between neurons within a limited range. As a consequence, the set of neurons that interacts in response to a given stimulus is defined not only by the given connectivity, but also by the spatial arrangements of inputs. One potential function of topographic feature maps is therefore to specify important functional interactions between neurons in a circuit. In the OB, chemotopy would favor mutual inhibition of MCs receiving similar inputs, which is expected to promote local sparsening and pattern decorrelation. We therefore propose that the chemotopic organization of activity patterns in the OB is an important feature that configures computational properties of the circuit even though it is not maintained during pattern processing.

## Materials and Methods

### Animals and odor stimulation.

Zebrafish were kept at 26–27 °C on a 13/11-h light/dark cycle under standard conditions. The transgenic line, HuC:YC [81], expressed the fluorescent protein, YC 2.1 [82], under the control of a fragment of the HuC promoter. In the adult zebrafish OB, HuC:YC is expressed selectively in MCs [54]. YC fluorescence did not change in response to odor stimulation and was used exclusively as an anatomical marker. No obvious differences were observed between MC odor responses in HuC:YC or wild-type fish recorded by loose-patch recordings, whole-cell recordings, or Ca^2+^ imaging.

Experiments were performed in an explant preparation of the intact brain, nose, and other sensory organs [32]. Briefly, adult (>3 mo old) zebrafish were cooled to 4 °C and decapitated in artificial cerebrospinal fluid [83]. After removal of eyes, jaws, and bones covering the ventral telencephalon, the preparation was placed in a flow chamber and slowly warmed up to room temperature. All animal procedures were performed in accordance with the animal care guidelines issued by the Federal Republic of Germany.

Odors were delivered through a constant flow directed at the ipsilateral inflow naris using a computer-controlled, pneumatically actuated high performance liquid chromatography (HPLC) injection valve (Rheodyne, http://www.rheodyne.com). From amino acids of the highest available purity (Fluka; Sigma-Aldrich, http://www.sigmaaldrich.com), frozen stock solutions (1 or 10 mM) were diluted to a final concentration of 10 μM immediately before the experiment. The minimum interstimulus interval was 90 s to avoid adaptation. The standard stimulus set included 16 amino acids used in previous studies [9,32,33] and a blank, which did not evoke responses.

### Temporally deconvolved Ca^2+^ imaging.

Neurons in the OB were loaded with the red-fluorescing Ca^2+^ indicator, rhod-2-AM, by bolus injection and imaged by two-photon microscopy as described [51]. Fluorescence imaging was performed with a custom two-photon microscope equipped with a mode-locked Ti:Sapphire laser (Mira900, 100 fs, 76 MHz; 830–850 nm; pumped by a 10-W Verdi laser; Coherent, http://www.coherent.com), a 20×, N.A. 0.95 objective lens (Olympus, http://www.olympus.co.jp/en/), and external detection optics. Rhod-2 and YC fluorescence were detected simultaneously in separate emission channels using bandpass filters of 515/30 nm and 610/75 nm, respectively. Laser intensity was adjusted to minimize photobleaching in each focal plane. Time series of rhod-2 images were converted to image series representing the relative change in fluorescence, *F,* in each pixel after stimulus onset (Δ*F/F*)

Image series in dataset 1 were acquired at 128 ms/frame and contained 128 × 256 pixels, covering a field of view of 163 × 81.5 μm^2^. Images in datasets 2 and 3 were acquired at 256 ms/frame and contained 256 × 256 pixels, covering 163 × 163 μm^2^. Time values represent the starting time of frames. The dataset to quantify the stability of responses (Figure S2) was acquired using the same parameters as in datasets 1 (MCs) or 2 (INs). *t* = 0 designates stimulus onset, which was determined as the first frame in which a response was observed. Because it is unclear when the response started within this frame, the first frame was omitted from analyses.

In each dataset, responses to the same sets of odors were collected in multiple optical sections at different *x, y,* and *z* positions throughout the volume of interest. The *z*-distance between focal planes that overlapped in *x* and *y* was 15 μm or greater. The average soma diameter of MCs is approximately 10 μm [32]; INs are smaller. Three-dimensional activity patterns were reconstructed from responses measured in 5–18 different optical sections in each dataset. In dataset 1, optical sections were distributed throughout the glomerular/MC layer in the ventrolateral OB. In datasets 2 and 3, optical sections also covered the central OB, where many granule cells are located. Dataset 1 is identical to the MC dataset described in [51]. The number of MCs recorded in each optical section was lower than that of INs because the density of MCs is much lower. Neuronal somata were outlined manually based on YC fluorescence (MCs) or rhod-2 fluorescence and Ca^2+^ signals (INs).

Firing-rate changes were reconstructed from Ca^2+^ signals by temporal deconvolution as described [51]. Parameters used for temporal deconvolution were τ_decay_ = 3 s and thr_noise_ = 1% for MCs, and τ_decay_ = 6 s and thr_noise_ = 1% for INs. These parameters are within the range yielding optimal reconstruction of firing-rate patterns, as determined previously [51]. As shown previously [51], differences in dye concentration or variability of parameters across neurons only minimally affects the reconstruction of population activity patterns.

### Data analysis.

Sparseness is a measure for the “peakiness” of a distribution that was derived by Vinje and Gallant [56] from a related measure developed by Rolls and Tovee [84]. The sparseness *S* was calculated as described [32,56] after setting negative values to zero: *S* = {1 − [(Σ*r*_n_/*N*)^2^/Σ(*r*_n_^2^/*N*)]}/[1 − (1/*N*)], where *r*_n_ is the nth response (response of the nth MC for population sparseness or response to the nth odor for sparseness of response profile) and *N* is the total number of responses (responses of different neurons to the same stimulus for population sparseness or responses to different stimuli for sparseness of response profiles). Sparseness is a value between zero and one and is independent of the number of responses *N* in the dataset.

To test whether the slow decrease in correlation between IN activity patterns (Figure 3A and 3B) is a consequence of the increasing population sparseness of IN activity (Figure 2C), we artificially increased the sparseness of IN response patterns. Random subsets of IN responses (up to 50%) were replaced by noise with an amplitude and distribution identical to the spontaneous activity of each IN. The sequence of correlation matrices remained similar after artificial sparsening, indicating that the slow decrease of correlation coefficients cannot be attributed to the increase in population sparseness.

Factor analysis was performed by a principal component analysis followed by varimax rotation and promax transformation, as described previously [9,32,62]. The number of factors was set to four based on results from correlation analysis and previous studies [9,32,33]. Factor dominance was quantified as described [32] and normalized onto the interval (0,1) by dividing by the maximum possible value (*I*_max_ = 0.1875 for four factors).

For the quantification of the rise time of excitatory responses, a sigmoid curve was fitted to the onset of each response. Rise time was then quantified as the time between 10% of the maximum in the fit and the peak time determined from the original trace. Inhibitory responses were not considered.

To estimate mean population firing rates of MCs and INs, all individual responses were averaged for each cell type and scaled to yield approximate mean population firing-rate changes. Scaling factors were determined previously by simultaneous TDCa imaging and electrophysiological recordings [51]. Because the TDCa signal reflects the firing-rate change relative to baseline, spontaneous firing rates, determined by electrophysiology in separate experiments, were added (MCs: 8.8 Hz [34]; INs: 0.7 Hz [B. Judkewitz; unpublished data]). The estimate of the mean population firing rate is sensitive to errors in determining the scaling factors. However, the time course of the estimated MC population firing rate was similar to that determined previously by electrophysiological recordings [32]. We therefore conclude that estimates of population firing rates based on TDCa imaging are valid, although precise measurements require extensive electrophysiological recordings.

The standard focality index was based on a similar index used previously [54]. Neurons with response intensities equal to or greater than 50% of the maximum response were selected from each activity pattern. This threshold is arbitrary and serves to exclude weakly active neurons that may contribute noise but are unlikely to have a substantial influence on focality. The distances between all pairs of selected neurons were weighted by the product of their response intensities (normalized so that the sum of all weights was one) and averaged, yielding <*d_selected_*>. The focality index *f* was calculated as 1 − (<*d_selected_*>/<*d_all_*>), where <*d_all_*> is the average distance between all neurons. This focality index *f* is related to the index used previously *f*_previous_ [54] by *f* = 1 − *f*_previous_; it is therefore more intuitive because low focalities are represented by values close to zero whereas extremely high focalities are represented by values close to one. The significance of focality was assessed by a statistical comparison of the measured focalities to focalities after randomly permuting the positions of all neurons.

In order to test whether results depend critically on the procedure used to quantify focality, we also tested a variety of modifications of the focality index. However, all procedures yielded similar results (Figure S4B–S4E), indicating that the quantification of focality using the standard index is robust.

## Supporting Information

Figure S1 Anatomical Relationship between MCs and Glomeruli in Zebrafish In 129 HuC:YC-positive neurons, primary dendrites were followed from the soma to glomeruli. Most MCs had only one primary dendrite. The histogram shows the distribution of distances between the centers of the MC soma and the associated glomerulus (mean ± SD: 30 ± 24 μm). Most MCs in zebrafish are therefore uniglomerular and closely associated with the innervated glomerulus. These results are consistent with a recent anatomical study [53] and indicate that zebrafish MCs differ anatomically from MCs in other teleost species such as the carp [85].
(31 KB PDF)

Figure S2 Stability of Odor Responses Average correlation matrices of activity patterns evoked by repeated stimulation with the same odor. While imaging a given focal plane, each of two stimuli (His, Tyr, Met, or Lys; 10 μM) was presented ten times in an interlaced fashion. Hence, 20 response patterns were measured at each focal plane, and the first and the last repetition of the same stimulus were separated by 17 intervening stimulations. Responses to the same sequence of stimuli were then measured in multiple focal planes in each OB. In total, responses were recorded from 179 MCs (*n* = 2 OBs; 90 ± 22 MCs per OB; mean ± SD) and 690 INs (*n* = 1 OB). For each OB and stimulus, the correlation matrix containing the pairwise correlation coefficients between the ten response patterns (raw Ca^2+^ signals, time-averaged over 2.4 s after response onset) was calculated. Figures show correlation matrices for MC and IN experiments, averaged over all stimuli and OBs (using the Fisher *Z* transformation). The average correlation coefficients between activity patterns evoked by repeated stimulation across MCs and INs were 0.84 ± 0.03 and 0.87 ± 0.03, respectively, indicating that measured responses of odor-evoked Ca^2+^ signals were stable.
(10 KB PDF)

Figure S3 Factor Analysis Distribution of factor loadings extracted from patterns of TDCa signals in successive time windows (datasets 1 and 2). Boxes in the first plot depict odors grouped by the dominance of the same factor. Labels describe the chemical property that is common to each group of odors and thus is represented by the corresponding factor.
Factor analysis extracts “principal” patterns called factors, each of which represents an elementary feature (or combination of features) common to a group of patterns. The feature represented by a factor usually corresponds to a chemical feature of the associated stimuli that has to be derived by visual inspection of the chemical structure [61,62]. Unlike principal components, factors are not necessarily orthogonal to each other. Factor loadings are a quantitative measure for the association of each stimulus-evoked pattern with each chemical feature and thus provide a natural basis for grouping of patterns by similarity. Individual activity patterns are closely associated with a group when one factor loading is dominant, i.e., when one loading is high and the others are close to zero. Grouping within a set of patterns is pronounced when most or all individual stimuli are dominated by a single factor loading.
(42 KB PDF)

Figure S4Further Analyses of Focality(A) Focality as a function of time for activity patterns with initial focalities equal to 0.4 or greater. Gray: time course for individual patterns; blue: average ± SD.(B–E) Comparison of different focality indices. The standard focality index (see Materials and Methods) was calculated as

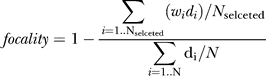
where *N* is the number of all cell pairs, *N_selected_,* is the number of cell pairs with TDCa signals greater than 50% of the maximum, *d_i_* is the distance between cells in pair *i,* and *w_i_* is the weight for cell pair *i*. The weight *w_i_* is calculated as the product of the TDCa signals of the cells in pair *i,* divided by the products of TDCa signals of all selected cell pairs so that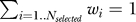 .
This procedure includes a threshold to exclude weakly active neurons (50% of maximum) and calculates the weights based on the pairwise products of activity levels. In order to test whether results depend critically on these procedures, we explored modifications of the standard focality index. The calculation of focality does not, in principle, require a threshold, but negative activity values are not allowed. We therefore recalculated the focality including all neurons with TDCa signals equal to zero or greater. In addition, we calculated the weights as the sum, rather than the product, of pairwise TDCa signals. Finally, we developed a modified focality index that includes also neurons with TDCa signals less than zero. In this index, raw weights *w*_raw_ were calculated as pairwise products or sums for all possible pairs of cells. Corrected weights *w* for each pair of cells were then calculated as *w* = −1/*w*_raw_ when the TDCa signal from one of the two cells in the corresponding pair was negative, or as *w* = *w*_raw_ otherwise. The focality index was then calculated using the corrected weights *w*.The figure shows results obtained with an index using no threshold (B and D), and an index including negative TDCa signals and using the sum to calculate weights (C and E). (B) and (C) show focalities of measured activity patterns as a function of time; (D) and (E) show focalities of factors as a function of time. Dots indicate significant differences (*p* < 0.05; Wilcoxon rank sum test) from the focality of randomized patterns. Absolute focality values differ somewhat from the standard index, as expected. In all cases, however, the time course of the focality was very similar to that observed with the standard index (Figures 6A and 11A). These results clearly show that the quantification of focality using the standard index is robust.(48 KB PDF)

Figure S5 Two-Dimensional Projections of Averaged Factors Extracted during the Initial Response Phase Factors representing distinct molecular features (long-chain, aromatic, and basic) were extracted from time-averaged activity patterns as in Figure 10 (0–768 ms for MCs and INs), binned in 20 × 20 × 20-μm^3^ voxels, centered on the centroid of the aromatic factor, averaged over animals, and projected into the horizontal plane. Crosshairs indicate the centroid of the aromatic factor. Note the segregation of activity representing different molecular features along the anterior–posterior axis.
(38 KB PDF)

Video S1 Topological Reorganization of Activity Patterns (Lys) Odor-evoked, spatiotemporal activity pattern across MCs and INs evoked by Lys (same experiment and conventions as in Figure 4A) covers the first second of the odor response. Data were acquired at 256 ms/frame and interpolated 4-fold. Hence, each frame corresponds to 64 ms. The speed is 8 frames/sec (~0.5 × real speed).
(2.5 MB AVI)

Video S2 Topological Reorganization of Activity Patterns (Trp) Odor-evoked, spatiotemporal activity pattern across MCs and INs evoked by Trp (same experiment as in Figure 4A). Conventions as in Video S1.
(2.4 MB AVI)

Video S3 Topological Reorganization of Activity Patterns (Ile) Odor-evoked, spatiotemporal activity pattern across MCs and INs evoked by Ile (same experiment as in Figure 4A). Conventions as in Video S1.
(2.3 MB AVI)

## Abbreviations

IN: interneuron
MC: mitral cell
OB: olfactory bulb
SD: standard deviation
TDCa imaging: temporally deconvolved Ca^2+^ imaging
YC: yellow cameleon

## References

[pbio-0050178-b001] Van Essen DC, Gallant JL (1994). Neural mechanisms of form and motion processing in the primate visual system. Neuron.

[pbio-0050178-b002] Konishi M, Takahashi TT, Wagner H, Sullivan WE, Carr CE, Edelman GM, Gall WE, Cowan WM (1988). Neurophysiological and anatomical substrates of sound localization in the owl. Auditory function: neurobiological bases of hearing.

[pbio-0050178-b003] Kaas JH (1997). Topographic maps are fundamental to sensory processing. Brain Res Bull.

[pbio-0050178-b004] Wilson RI, Mainen ZF (2006). Early events in olfactory processing. Annu Rev Neurosci.

[pbio-0050178-b005] Araneda RC, Kini AD, Firestein S (2000). The molecular receptive range of an odorant receptor. Nature Neurosci.

[pbio-0050178-b006] Stewart WB, Kauer JS, Shepherd GM (1979). Functional organization of rat olfactory bulb analysed by the 2-deoxyglucose method. J Comp Neurol.

[pbio-0050178-b007] Rubin BD, Katz LC (1999). Optical imaging of odorant representations in the mammalian olfactory bulb. Neuron.

[pbio-0050178-b008] Wachowiak M, Cohen LB (2001). Representation of odorants by receptor neuron input to the mouse olfactory bulb. Neuron.

[pbio-0050178-b009] Friedrich RW, Korsching SI (1997). Combinatorial and chemotopic odorant coding in the zebrafish olfactory bulb visualized by optical imaging. Neuron.

[pbio-0050178-b010] Friedrich RW, Korsching SI (1998). Chemotopic, combinatorial and noncombinatorial odorant representations in the olfactory bulb revealed using a voltage-sensitive axon tracer. J Neurosci.

[pbio-0050178-b011] Leon M, Johnson BA (2003). Olfactory coding in the mammalian olfactory bulb. Brain Res Brain Res Rev.

[pbio-0050178-b012] Uchida N, Takahashi YK, Tanifuji M, Mori K (2000). Odor maps in the mammalian olfactory bulb: Domain organization and odorant structural features. Nat Neurosci.

[pbio-0050178-b013] Mori K, Takahashi YK, Igarashi KM, Yamaguchi M (2006). Maps of odorant molecular features in the mammalian olfactory bulb. Physiol Rev.

[pbio-0050178-b014] Mombaerts P (2001). How smell develops. Nat Rev Neurosci.

[pbio-0050178-b015] Hallem EA, Ho MG, Carlson JR (2004). The molecular basis of odor coding in the *Drosophila* antenna. Cell.

[pbio-0050178-b016] Malnic B, Hirono J, Sato T, Buck LB (1999). Combinatorial receptor codes for odors. Cell.

[pbio-0050178-b017] Takahashi YK, Kurosaki M, Hirono S, Mori K (2004). Topographic representation of odorant molecular features in the rat olfactory bulb. J Neurophysiol.

[pbio-0050178-b018] Belluscio L, Katz LC (2001). Symmetry, stereotypy, and topography of odorant representations in mouse olfactory bulbs. J Neurosci.

[pbio-0050178-b019] Fuss SH, Korsching SI (2001). Odorant feature detection: Activity mapping of structure response relationships in the zebrafish olfactory bulb. J Neurosci.

[pbio-0050178-b020] Xu F, Liu N, Kida I, Rothman DL, Hyder F (2003). Odor maps of aldehydes and esters revealed by functional MRI in the glomerular layer of the mouse olfactory bulb. Proc Natl Acad Sci U S A.

[pbio-0050178-b021] Friedrich RW, Stopfer M (2001). Recent dynamics in olfactory population coding. Curr Opin Neurobiol.

[pbio-0050178-b022] Andres KH, Wolstenholme GEW, Knight J (1970). Anatomy and ultrastructure of the olfactory bulb in fish, amphibia, reptiles, birds and mammals. Ciba foundation symposium on taste and smell in vertebrates.

[pbio-0050178-b023] Allison AC (1953). The morphology of the olfactory system in the vertebrates. Biol Rev.

[pbio-0050178-b024] Aungst JL, Heyward PM, Puche AC, Karnup SV, Hayar A (2003). Centre-surround inhibition among olfactory bulb glomeruli. Nature.

[pbio-0050178-b025] Shepherd GM, Chen WR, Greer CA, Shepherd GM (2004). Olfactory bulb. The synaptic organization of the brain.

[pbio-0050178-b026] Lowe G (2003). Electrical signaling in the olfactory bulb. Curr Opin Neurobiol.

[pbio-0050178-b027] Adrian ED (1950). The electrical activity of the mammalian olfactory bulb. EEG Clin Neurophysiol.

[pbio-0050178-b028] Macrides F, Chorover SL (1972). Olfactory bulb units: Activity correlated with inhalation cycles and odor quality. Science.

[pbio-0050178-b029] Meredith M (1986). Patterned response to odor in mammalian olfactory bulb: The influence of intensity. J Neurophysiol.

[pbio-0050178-b030] Gray CM, Skinner JE (1988). Centrifugal regulation of neuronal activity in the olfactory bulb of the waking rabbit as revealed by reversible cryogenic blockade. Exp Brain Res.

[pbio-0050178-b031] Buonviso N, Chaput MA, Berthommier F (1992). Temporal pattern analyses in pairs of neighboring mitral cells. J Neurophysiol.

[pbio-0050178-b032] Friedrich RW, Laurent G (2001). Dynamic optimization of odor representations in the olfactory bulb by slow temporal patterning of mitral cell activity. Science.

[pbio-0050178-b033] Friedrich RW, Habermann CJ, Laurent G (2004). Multiplexing using synchrony in the zebrafish olfactory bulb. Nature Neurosci.

[pbio-0050178-b034] Friedrich RW, Laurent G (2004). Dynamics of olfactory bulb input and output activity during odor stimulation in zebrafish. J Neurophysiol.

[pbio-0050178-b035] Imamura K, Mataga N, Mori K (1992). Coding of odor molecules by mitral/tufted cells in rabbit olfactory bulb. I. Aliphatic compounds. J Neurophysiol.

[pbio-0050178-b036] Katoh K, Koshimoto H, Tani A, Mori K (1993). Coding of odor molecules by mitral/tufted cells in rabbit olfactory bulb. II. Aromatic compounds. J Neurophysiol.

[pbio-0050178-b037] Rinberg D, Koulakov A, Gelperin A (2006). Sparse odor coding in awake behaving mice. J Neurosci.

[pbio-0050178-b038] Guthrie KM, Anderson AJ, Leon M, Gall C (1993). Odor-induced increases in c-fos mRNA expression reveal an anatomical “unit” for odor processing in olfactory bulb. Proc Natl Acad Sci U S A.

[pbio-0050178-b039] Sallaz M, Jourdan F (1993). C-fos expression and 2-deoxyglucose uptake in the olfactory bulb of odour-stimulated awake rats. Neuroreport.

[pbio-0050178-b040] Sallaz M, Jourdan F (1996). Odour-induced c-fos expression in the rat olfactory bulb: Involvement of centrifugal afferents. Brain Res.

[pbio-0050178-b041] Edwards JG, Michel WC (2002). Odor-stimulated glutamatergic neurotransmission in the zebrafish olfactory bulb. J Comp Neurol.

[pbio-0050178-b042] Yang X, Renken R, Hyder F, Siddeek M, Greer CA (1998). Dynamic mapping at the laminar level of odor-elicited responses in rat olfactory bulb by functional MRI. Proc Natl Acad Sci U S A.

[pbio-0050178-b043] Cinelli AR, Hamilton KA, Kauer JS (1995). Salamander olfactory bulb neuronal activity observed by video rate, voltage-sensitive dye imaging. III. Spatial and temporal properties of responses evoked by odorant stimulation. J Neurophysiol.

[pbio-0050178-b044] Helmchen F, Denk W (2005). Deep tissue two-photon microscopy. Nat Methods.

[pbio-0050178-b045] Denk W, Strickler JH, Webb WW (1990). Two-photon laser scanning fluorescence microscopy. Science.

[pbio-0050178-b046] Stosiek C, Garaschuk O, Holthoff K, Konnerth A (2003). In vivo two-photon calcium imaging of neuronal networks. Proc Natl Acad Sci U S A.

[pbio-0050178-b047] Brustein E, Marandi N, Kovalchuk Y, Drapeau P, Konnerth A (2003). “In vivo” monitoring of neuronal network activity in zebrafish by two-photon Ca^2+^ imaging. Pflügers Arch.

[pbio-0050178-b048] Kerr JN, Greenberg D, Helmchen F (2005). Imaging input and output of neocortical networks in vivo. Proc Natl Acad Sci U S A.

[pbio-0050178-b049] Ohki K, Chung S, Ch'ng YH, Kara P, Reid RC (2005). Functional imaging with cellular resolution reveals precise micro-architecture in visual cortex. Nature.

[pbio-0050178-b050] Ohki K, Chung S, Kara P, Hubener M, Bonhoeffer T (2006). Highly ordered arrangement of single neurons in orientation pinwheels. Nature.

[pbio-0050178-b051] Yaksi E, Friedrich RW (2006). Reconstruction of firing rate changes across neuronal populations by temporally deconvolved Ca^2+^ imaging. Nat Methods.

[pbio-0050178-b052] Byrd CA, Brunjes PC (1995). Organization of the olfactory system in the adult zebrafish: histological, immunohistochemical, and quantitative analysis. J Comp Neurol.

[pbio-0050178-b053] Fuller CL, Yettaw HK, Byrd CA (2006). Mitral cells in the olfactory bulb of adult zebrafish *(Danio rerio):* Morphology and distribution. J Comp Neurol.

[pbio-0050178-b054] Li J, Mack JA, Souren M, Yaksi E, Higashijima S (2005). Early development of functional spatial maps in the zebrafish olfactory bulb. J Neurosci.

[pbio-0050178-b055] Carr WES, Atema J, Fay RR, Popper AN, Tavolga WN (1988). The molecular nature of chemical stimuli in the aquatic environment. Sensory biology of aquatic animals.

[pbio-0050178-b056] Vinje WE, Gallant JL (2000). Sparse coding and decorrelation in primary visual cortex during natural vision. Science.

[pbio-0050178-b057] Uchida N, Mainen ZF (2003). Speed and accuracy of olfactory discrimination in the rat. Nat Neurosci.

[pbio-0050178-b058] Abraham NM, Spors H, Carleton A, Margrie TW, Kuner T (2004). Maintaining accuracy at the expense of speed: Stimulus similarity defines odor discrimination time in mice. Neuron.

[pbio-0050178-b059] Rinberg D, Koulakov A, Gelperin A (2006). Speed-accuracy tradeoff in olfaction. Neuron.

[pbio-0050178-b060] Friedrich RW (2006). Mechanisms of odor discrimination: Neurophysiological and behavioral approaches. Trends Neurosci.

[pbio-0050178-b061] Bieber SL, Smith DV (1986). Multivariate analysis of sensory data: A comparison of methods. Chem Senses.

[pbio-0050178-b062] Reyment R, Jöreskog KG (1996). Applied factor analysis in the natural sciences. 2nd edition.

[pbio-0050178-b063] Murphy GJ, Glickfeld LL, Balsen Z, Isaacson JS (2004). Sensory neuron signaling to the brain: Properties of transmitter release from olfactory nerve terminals. J Neurosci.

[pbio-0050178-b064] Rhein LD, Cagan RH (1983). Biochemical studies of olfaction: Binding specificity of odorants to a cilia preparation from rainbow trout olfactory rosettes. J Neurochem.

[pbio-0050178-b065] Caprio J, Byrd RJ (1984). Electrophysiological evidence for acidic, basic, and neutral amino acid olfactory receptor sites in the catfish. J Gen Physiol.

[pbio-0050178-b066] Ng M, Roorda RD, Lima SQ, Zemelman BV, Morcillo P (2002). Transmission of olfactory information between three populations of neurons in the antennal lobe of the fly. Neuron.

[pbio-0050178-b067] Wilson RI, Turner GC, Laurent G (2004). Transformation of olfactory representations in the *Drosophila* antennal lobe. Science.

[pbio-0050178-b068] Egger V, Svoboda K, Mainen ZF (2005). Dendrodendritic synaptic signals in olfactory bulb granule cells: Local spine boost and global low-threshold spike. J Neurosci.

[pbio-0050178-b069] Murphy GJ, Darcy DP, Isaacson JS (2005). Intraglomerular inhibition: Signaling mechanisms of an olfactory microcircuit. Nat Neurosci.

[pbio-0050178-b070] Pressler RT, Strowbridge BW (2006). Blanes cells mediate persistent feedforward inhibition onto granule cells in the olfactory bulb. Neuron.

[pbio-0050178-b071] Cang J, Isaacson JS (2003). In vivo whole-cell recording of odor-evoked synaptic transmission in the rat olfactory bulb. J Neurosci.

[pbio-0050178-b072] Wellis DP, Scott JW (1990). Intracellular responses of identified rat olfactory bulb interneurons to electrical and odor stimulation. J Neurophysiol.

[pbio-0050178-b073] Luo M, Katz LC (2001). Response correlation maps of neurons in the mammalian olfactory bulb. Neuron.

[pbio-0050178-b074] Hall BJ, Delaney KR (2002). Contribution of a calcium-activated non-specific conductance to NMDA receptor-mediated synaptic potentials in granule cells of the frog olfactory bulb. J Physiol.

[pbio-0050178-b075] Margrie TW, Schaefer AT (2003). Theta oscillation coupled spike latencies yield computational vigour in a mammalian sensory system. J Physiol.

[pbio-0050178-b076] Kapoor V, Urban NN (2006). Glomerulus-specific, long-latency activity in the olfactory bulb granule cell network. J Neurosci.

[pbio-0050178-b077] Shang Y, Claridge-Chang A, Sjulson L, Pypaert M, Miesenbock G (2007). Excitatory local circuits and their implications for olfactory processing in the fly antennal lobe. Cell.

[pbio-0050178-b078] Didier A, Carleton A, Bjaalie JG, Vincent JD, Ottersen OP (2001). A dendrodendritic reciprocal synapse provides a recurrent excitatory connection in the olfactory bulb. Proc Natl Acad Sci U S A.

[pbio-0050178-b079] Isaacson JS (1999). Glutamate spillover mediates excitatory transmission in the rat olfactory bulb. Neuron.

[pbio-0050178-b080] Døving KB, Selset R, Thommesen G (1980). Olfactory sensitivity to bile acids in salmonid fishes. Acta Physiol Scand.

[pbio-0050178-b081] Higashijima S, Masino MA, Mandel G, Fetcho JR (2003). Imaging neuronal activity during zebrafish behavior with a genetically encoded calcium indicator. J Neurophysiol.

[pbio-0050178-b082] Miyawaki A, Llopis J, Heim R, McCaffery JM, Adams JA (1997). Fluorescent indicators for Ca^2+^ based on green fluorescent proteins and calmodulin. Nature.

[pbio-0050178-b083] Mathieson WB, Maler L (1988). Morphological and electrophysiological properties of a novel in vitro preparation: The electrosenspry lateral line lobe brain slice. J Comp Physiol A.

[pbio-0050178-b084] Rolls ET, Tovee MJ (1995). Sparseness of the neuronal representation of stimuli in the primate temporal visual-cortex. J Neurophysiol.

[pbio-0050178-b085] Satou M (1990). Synaptic organization, local neuronal circuitry, and functional segregation of the teleost olfactory bulb. Prog Neurobiol.

