# Supplementary material for: Topological Reorganization of Odor Representations in the Olfactory Bulb
Source: PLoS Biol. 2007 Jul 3;5(7):e178. doi: 10.1371/journal.pbio.0050178 (PMC1904499; doi:10.1371/journal.pbio.0050178)
Supplement: Supplementary file 1 [file pbio.0050178.sg001.pdf]

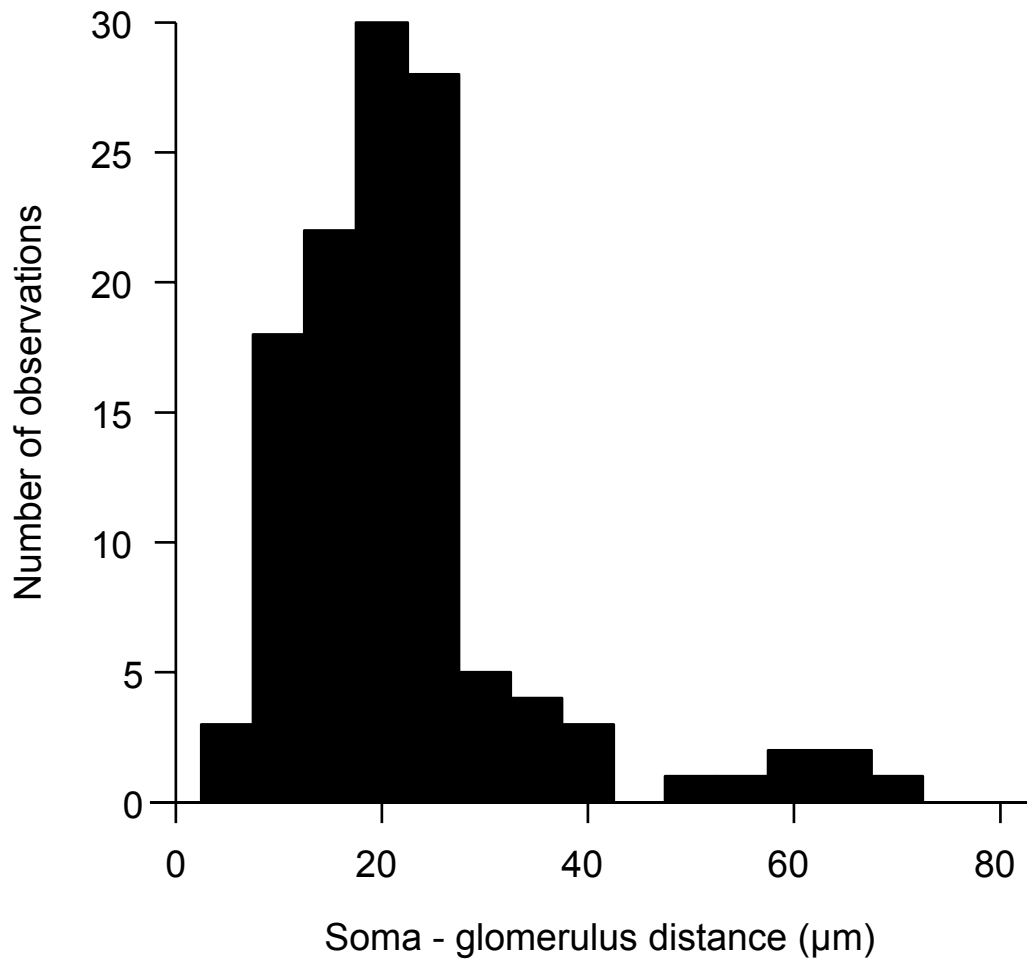
Number of observations
30
25
20
15
10
5
0
0
20
40
60
80
Soma - glomerulus distance (µm)
