# Supplementary material for: Topological Reorganization of Odor Representations in the Olfactory Bulb
Source: PLoS Biol. 2007 Jul 3;5(7):e178. doi: 10.1371/journal.pbio.0050178 (PMC1904499; doi:10.1371/journal.pbio.0050178)
Supplement: Supplementary file 2 [file pbio.0050178.sg002.pdf]

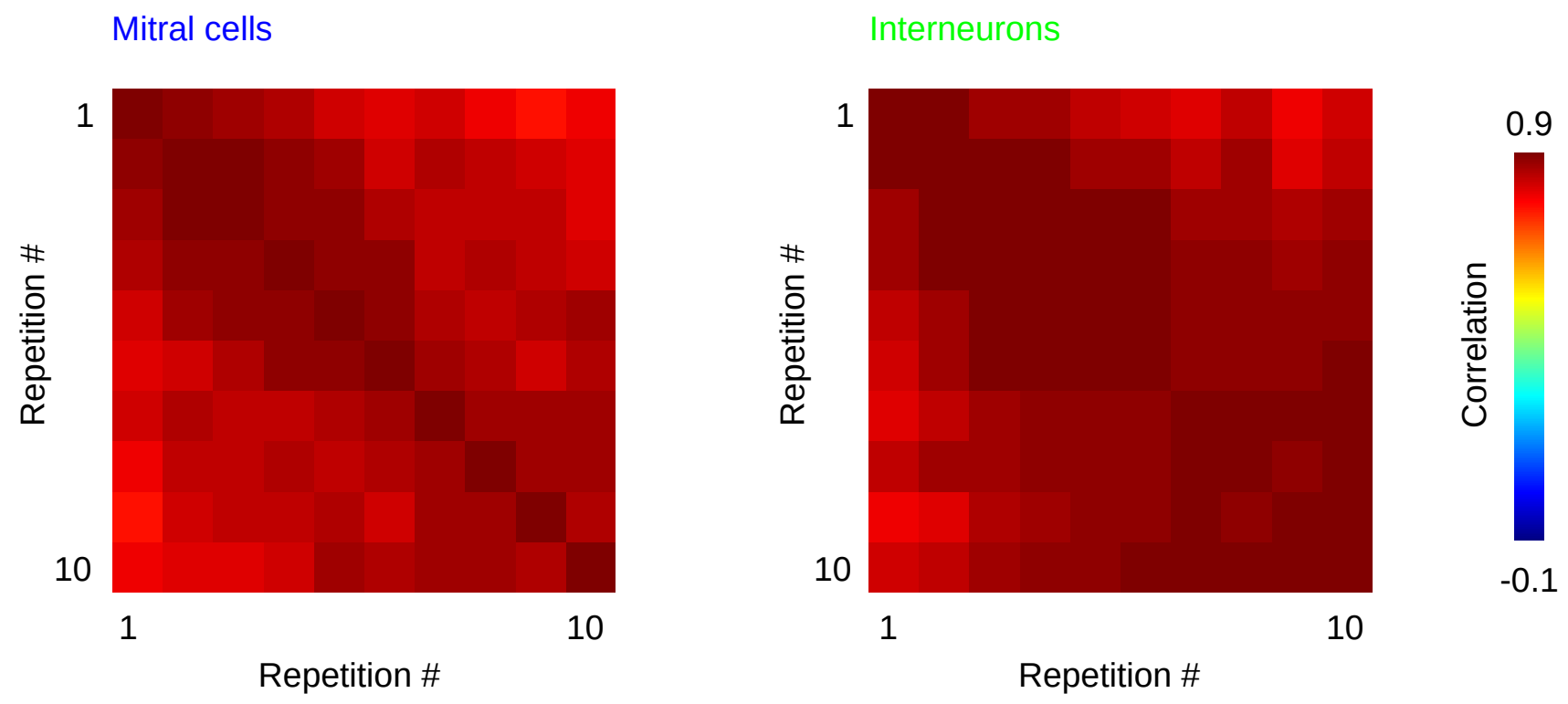
Mitral cells
Interneurons
1
10
Repetition #
1
10
Repetition #
0.9
-0.1
Correlation
