# Supplementary material for: Topological Reorganization of Odor Representations in the Olfactory Bulb
Source: PLoS Biol. 2007 Jul 3;5(7):e178. doi: 10.1371/journal.pbio.0050178 (PMC1904499; doi:10.1371/journal.pbio.0050178)
Supplement: Supplementary file 3 [file pbio.0050178.sg003.pdf]

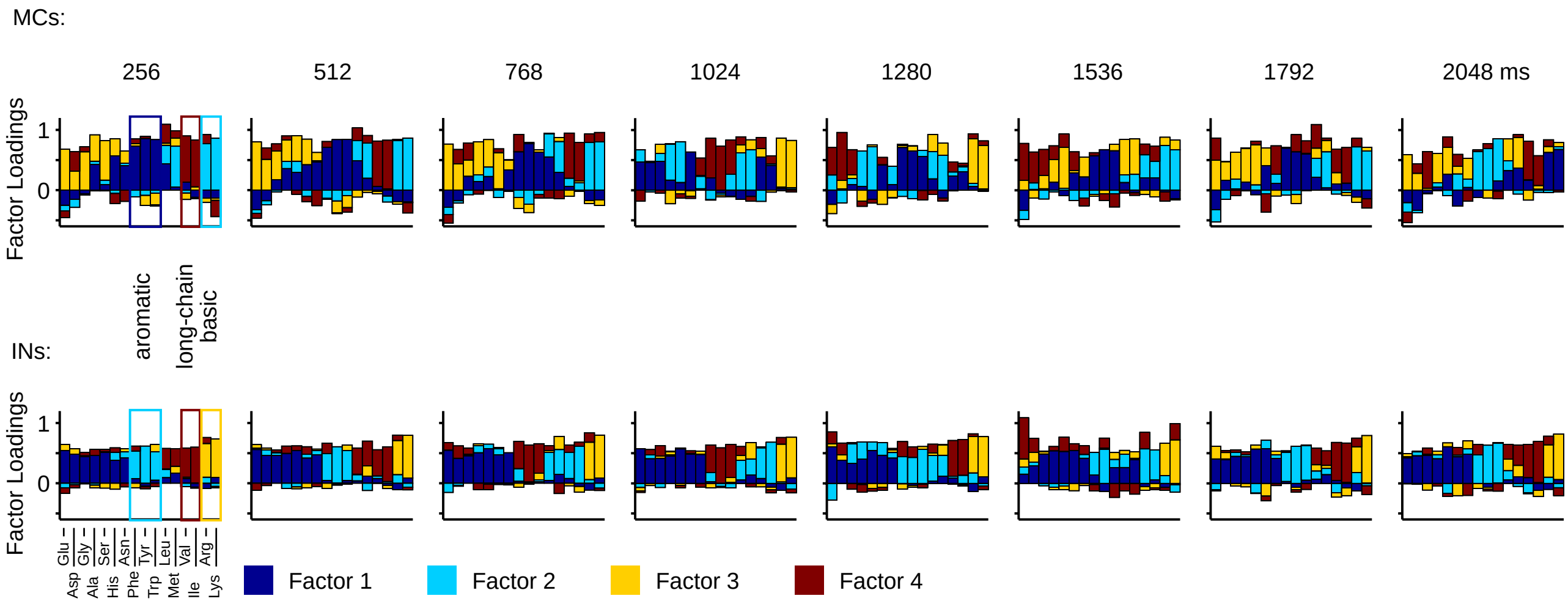

MCs:
256
512
768
1024
1280
1536
1792
2048 ms
Factor Loadings
1
0
aromatic
long-chain
basic
INs:
Factor Loadings
1
0
Glu
Asp
Gly
Ala
Ser
His
Asn
Phe
Tyr
Trp
Leu
Met
Val
Ile
Arg
Lys
Factor 1
Factor 2
Factor 3
Factor 4
