# Supplementary material for: Topological Reorganization of Odor Representations in the Olfactory Bulb
Source: PLoS Biol. 2007 Jul 3;5(7):e178. doi: 10.1371/journal.pbio.0050178 (PMC1904499; doi:10.1371/journal.pbio.0050178)
Supplement: Supplementary file 4 [file pbio.0050178.sg004.pdf]

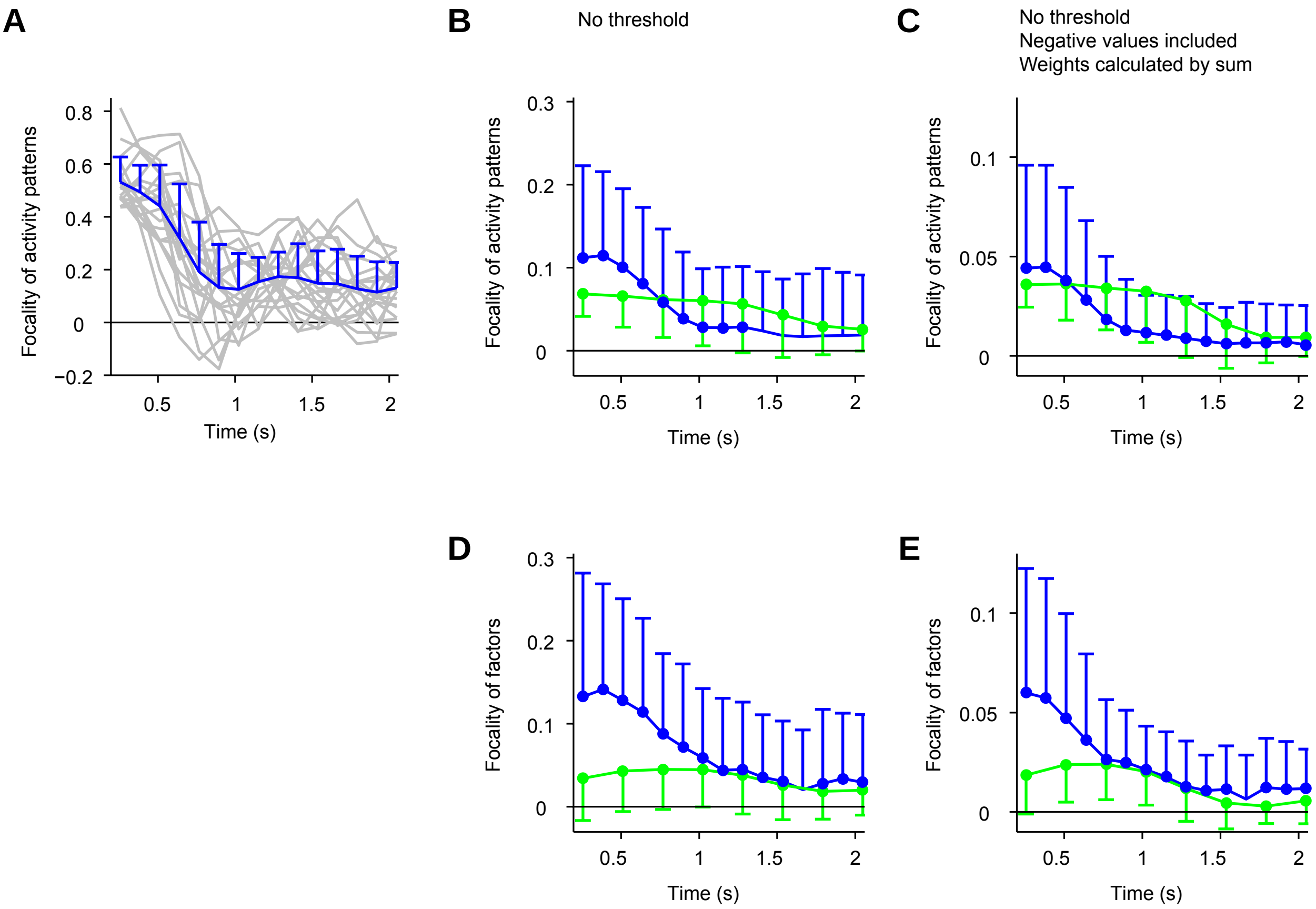
A
B
No threshold
C
No threshold
Negative values included
Weights calculated by sum
D
E
Focality of activity patterns
Focality of factors
Time (s)
0.8
0.6
0.4
0.2
0
−0.2
0.3
0.1
0.05
0.5
1
1.5
2
