# Supplementary material for: Topological Reorganization of Odor Representations in the Olfactory Bulb
Source: PLoS Biol. 2007 Jul 3;5(7):e178. doi: 10.1371/journal.pbio.0050178 (PMC1904499; doi:10.1371/journal.pbio.0050178)
Supplement: Supplementary file 5 [file pbio.0050178.sg005.pdf]

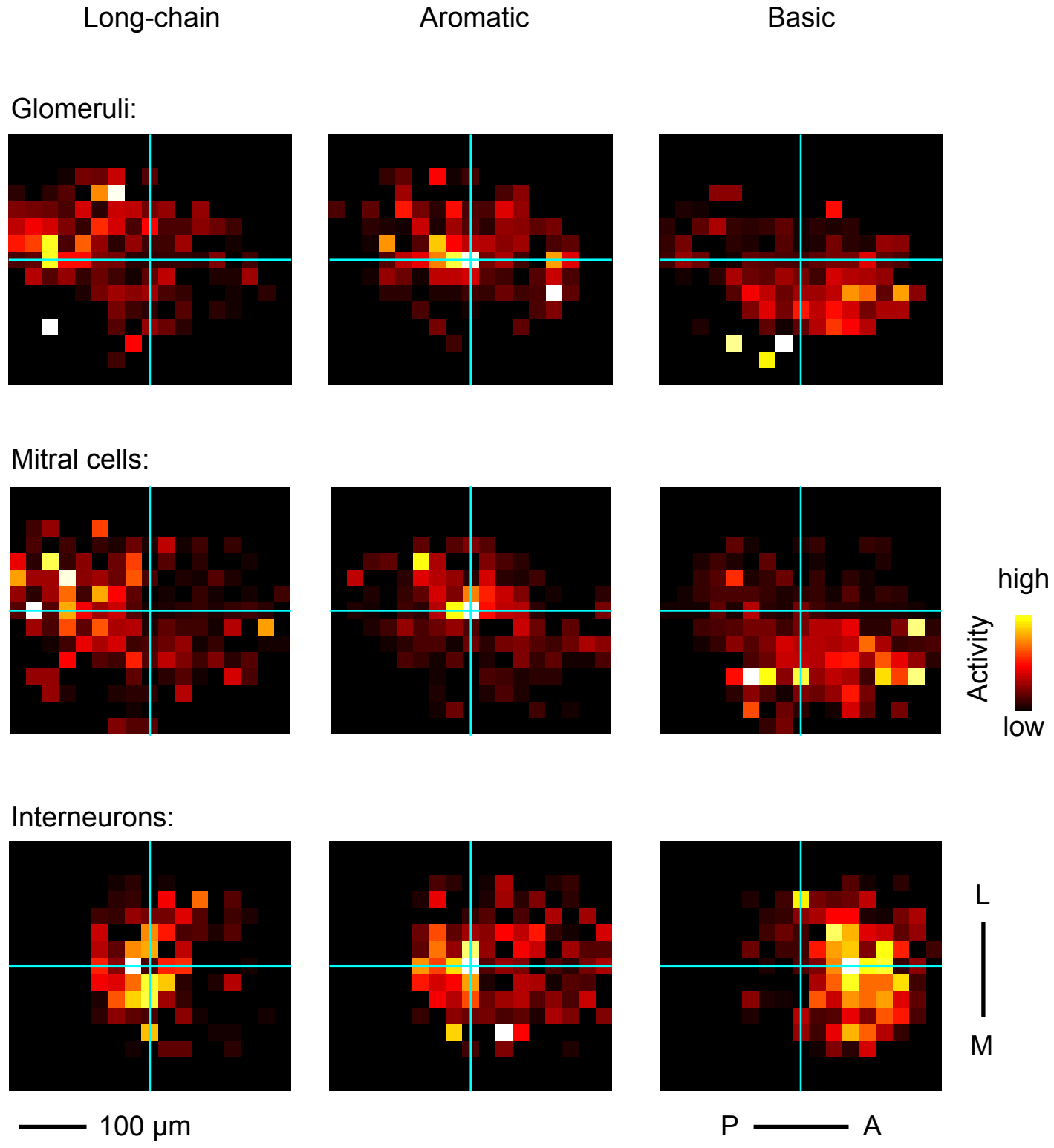
Long-chain
Aromatic
Basic
Glomeruli:
Mitral cells:
high
Activity
low
Interneurons:
L
M
100 µm
P
A
